# Is Continuous Monitoring of Skin Surface Temperature a Reliable Proxy to Assess the Thermoregulatory Response in Endurance Horses During Field Exercise?

**DOI:** 10.3389/fvets.2022.894146

**Published:** 2022-05-27

**Authors:** Elisabeth-Lidwien J. M. M. Verdegaal, Gordon S. Howarth, Todd J. McWhorter, Catherine J. G. Delesalle

**Affiliations:** ^1^Equine Health and Performance Centre, University of Adelaide, Adelaide, SA, Australia; ^2^Department of Translational Physiology, Infectiology and Public Health, Research Group of Comparative Physiology, Faculty of Veterinary Medicine, Ghent University, Merelbeke, Belgium; ^3^Gastroenterology Department, Women's and Children's Hospital, Adelaide, SA, Australia

**Keywords:** skin surface temperature, thermoregulation, metabolic heat, endurance, exercise, performance, competition, hyperthermia

## Abstract

Hyperthermia is a performance and welfare issue for exercising horses. The thermoregulatory stressors associated with exercise have typically been estimated by responses in the laboratory. However, monitoring surface skin temperature (T_*sk*_) coincident with core temperature (T_*c*_) has not previously been investigated in horses exercising in the field. We investigated the suitability of monitoring surface T_*sk*_ as a metric of the thermoregulatory response, and simultaneously investigated its relationship with T_*c*_ using gastrointestinal (GI) temperature. We evaluated T_*sk*_ in 13 endurance horses competing during four endurance rides over 40 km (*n* = 1) or a total of 80 km (*n* = 12) distance. Following each 40-km loop, the horses were rested for 60 min. T_*sk*_ and T_*c*_ were continuously recorded every 15 s by an infrared thermistor sensor located in a modified belt and by telemetric GI pill, respectively, and expressed as mean ± SD. The net area under the curve (AUC) was calculated to estimate the thermoregulatory response to the thermal load of T_*sk*_ over time (°C × minutes) using the trapezoidal method. The relationship between T_*sk*_ and T_*c*_ was assessed using scatterplots, paired *t*-test or generalized linear model ANOVA (delta T_*sk*_) (*n* = 8). Ambient temperature ranged from 6.7°C to 18.4°C. No relationship was found between T_*sk*_ and T_*c*_ profiles during exercise and recovery periods, and no significant difference between delta T_*sk*_ results was detected when comparing exercise and rest. However, time to maximum T_*sk*_ (67 min) was significantly reduced compared to T_*c*_ (139 min) (*p* = 0.0004) with a significantly lesser maximum T_*sk*_ (30.3°C) than T_*c*_ (39°C) (*p* = 0.0002) during exercise. Net AUC T_*sk*_ was 1,164 ± 1,448 and −305 ± 388°C × minutes during periods of exercise and recovery, respectively. We conclude that T_*sk*_ monitoring does not provide a reliable proxy for the thermoregulatory response and horse welfare, most probably because many factors can modulate T_*sk*_ without directly affecting T_*c*_. Those factors, such as weather conditions, applicable to all field studies can influence the results of T_*sk*_ in endurance horses. The study also reveals important inter-individual differences in T_*sk*_ and T_*c*_ time profiles, emphasizing the importance of an individualized model of temperature monitoring.

## Introduction

In the face of climate change, hyperthermia and heat stress have become increasingly challenging issues for a wide array of equine sports disciplines, especially during field competitions (1, 2). An increase in core body temperature (T_*c*_) leading to hyperthermia may cause widespread cytotoxicity as a direct effect of heat, while indirect effects related to decreased cardiac output cause neural and intestinal ischemia. If unchecked, these systemic inflammation processes eventually lead to exertional heat illness (EHI) (3–5). The clinical manifestations of EHI include neurological signs, varying from irritability, depression, ataxia, collapse, and may further progress to exertional heat stroke (EHS) with multi-organ dysfunction and death (3–5). In human athletes, EHS is among the top three causes of sudden death, and, in summer, it is the number one cause of athlete death in the USA (3). Similarly, both EHI and EHS are problematic conditions in equine athletes (6). The prevalence of metabolic disorders in, for example, endurance horses, triggered by thermoregulatory-induced physiological feedback failure and exhaustion, ranges from 4.2% to 15% (7–14). Recently, the prevalence of EHI in racehorses has been reported: two studies from Japan state prevalence of 0.09% during summer with a clear increase over the past few years (15, 16). Moreover, a study in Eastern Australia focused on selected EHI cases post-exercise at the racetrack and suggested an EHI incidence of up to 9.5% during hot summer months (17). The latter study used the four severity levels of EHI reported by Brownlow et al. (4) and concluded that 96% of horses could be categorized as Level 1. This suggests that a low level and thus discrete EHI cases may have been overlooked in the past.

Environmental conditions are the dominant risk factor in heat stress events, and EHI cases are expected to further increase in prevalence due to global warming (1, 4, 15–17). That worrying reality drives the ongoing efforts of research groups worldwide to develop reliable approaches to monitor and safeguard thermoregulatory wellbeing in horses (2, 18–21). We have previously reported on the continuous monitoring of the thermoregulatory response in endurance and trotter horses using a telemetric gastrointestinal (GI) pill (2). Briefly, the GI temperature pill is a non-invasive method to monitor T_*c*_ during exercise in the field. The GI pill was administered the night before the endurance competition to allow recording of a large temperature data set to establish T_*c*_ profiles of exercising horses. The data were telemetrically transferred to a device located in a belt under the saddle girth and supported continuous real-time monitoring. It was demonstrated that the GI telemetry pill is a reliable method to monitor T_*c*_ and assess the individual dynamic thermal response in exercising horses in the field. The study revealed important inter-individual differences in T_*c*_ time profiles, despite the horses performing the same exercise protocol. This finding emphasizes the importance of an individualized model of temperature monitoring. It can be concluded that the continuous monitoring approach allows for intervention at the early stages of heat accumulation and the possibility to take prompt and effective preventative measures.

Another elegant approach to monitoring the thermoregulatory response in exercising horses in the field could be continuous monitoring of surface skin temperature (T_*sk*_) as a reliable proxy for monitoring thermoregulatory wellbeing. Advantages of using tools, such as infrared thermography (IRT), to assess T_*sk*_ include the non-invasive nature and easy collection of temperature data (22–24). Notably, most exercise studies use such monitoring methods to assess T_*sk*_ only pre- or post-exercise, not during exercise. To illustrate, a recent systematic review of T_*sk*_ studies performed in human endurance athletes has reported that only a few studies involved the continuous monitoring of T_*sk*_; only two out of the 45 exercise studies monitored T_*sk*_ every 30 s, one study every 180 s, and four studies every 5 min (24). Also, these human studies highlight the existence of essential inter-individual differences with respect to the T_*sk*_ response time profile (24). Important inter-individual T_*sk*_ differences have been reported in all mammals (23, 25). For example, an equine study comparing T_*sk*_ in 21 horses at rest revealed significant inter-individual differences (26). Most existing equine thermoregulatory exercise studies (*n* = 12 studies) involving T_*sk*_ have focused on single point post-exercise measurements using a handheld IRT camera, leaving both the pre-exercise and intra-exercise periods out of their scope (21, 22, 27–37). An overview of all T_*sk*_ studies on horses at rest and during exercise is presented in Supplementary Table 1.

To date, only a few equine studies have focused on continuous monitoring of T_*sk*_ during exercise and recovery (*n* = 7, Supplementary Table 1). All were conducted in laboratory conditions, for example, on a treadmill (37–42). Only one study has recorded T_*sk*_ continuously during a short bout of field exercise of 4.5-min duration to investigate the effect of pre-exercise cooling in 10 horses (20). The study recorded surface T_*sk*_ using a microchip (i-Button®) attached to the skin with removable glue and simultaneously monitored rectal temperature (T_*re*_) using a rectal probe; both T_*sk*_ and T_*re*_ reduced over time (3°C and 0.3°C, respectively).

From a physiological standpoint, it is essential to appreciate that a time lag exists between exercise-induced metabolic heat (MH) output and T_*c*_ evolvement. The T_*c*_ is subsequently translated into an additional temperature time-lag evolvement, expressed at several different anatomical locations, such as the rectum, the muscular compartment, and the skin surface, whether or not additionally complicated by environmental factors, such as hot and humid weather (1, 25, 39, 43–48). Most importantly, T_*re*_ evolvement has been reported to significantly lag behind the T_*c*_ both during and after exercise (19, 38, 49), which renders the T_*re*_ less suitable as a “whistle blower” for thermoregulatory instability. In our previous studies, we demonstrated that GI temperature is a more reliable proxy for the thermoregulatory response and T_*c*_ when compared to T_*re*_, and that continuously monitoring GI temperature evolvement demonstrated how the equine body copes with exercise, challenging the thermoregulatory system (2, 19). Endurance horses, for example, reached their mean maximum T_*c*_ (39.0 ± 0.4°C) during exercise at 75% of completion of exercise, and T_*c*_ returned to the baseline within 60 min into recovery (2). However, the mean T_*c*_ was still 38.8 ± 0.4°C at a heart rate (HR) of 60 bpm, which currently governs “fit-to-continue” competition decisions (50), thus questioning the use of HR values to make such important decisions. However, contrary to this finding, trotter horses reached a comparable mean maximum T_*c*_ (38.8 ± 0.5°C) during recovery. Moreover, in 30% of trotters, T_*c*_ was still > 39°C at the end of the recovery period (40 ± 32 min), following exercise in a cool environment, findings that may have post-exercise management implications.

To identify a reliable proxy for thermoregulatory response in the field, a solid correlation must exist between that specific proxy and T_*c*_ evolvement, despite the existence of a time lag (24, 47). However, currently, very few equine studies have involved the simultaneous continuous monitoring of T_*c*_ (either using carotid artery temperature, or a GI pill, or the T_*re*_), together with an additional temperature monitoring device during field exercise (19, 20, 51). On the other hand, with the ongoing development of new wearables and sensors, there are an increasing number of exercise studies investigating continuous T_*sk*_, monitoring wearables (20, 37–40, 42, 52) (Supplementary Table 1 in Supplementary Material). These devices all provide data output, although the physiological meaning of these data is not always clear.

The MH produced during exercise needs to be dissipated from the horse to the surrounding environment through four main pathways, namely, radiation, conduction, convection, and evaporation, the last being the most essential and pivotal pathway in horses (23, 48, 53–56). Evaporation from the body surface is mainly achieved by increased blood flow, cutaneous vasodilation followed by evaporation of sweat from the skin (70–85% of the MH load) (25, 48, 54, 56–59). Heat loss by evaporation can be enhanced by cooling techniques (35, 40, 43). When focusing on T_*sk*_ as a temperature monitoring method, it is vital to keep in mind that all these pathways to dissipate MH to the environment may influence the T_*sk*_ data output.

Monitoring T_*sk*_ simultaneously with T_*c*_ using the GI temperature pill during field exercise has not yet been investigated. The relationship between T_*sk*_ and T_*c*_ is not well understood due to physiological, endocrine, or vasomotor influences on both temperatures (25, 56, 59). Some studies have tried to correlate both T_*c*_ and T_*sk*_ (39–42). The current study aimed to evaluate the usefulness of continuous monitoring of T_*sk*_ by means of a surface IR sensor device as a proxy for the thermoregulatory response. For this purpose, the T_*sk*_ relationship with T_*c*_ was investigated by simultaneous and continuous telemetric measurements during real-time field competitions under cool weather conditions. Endurance horses were equipped with several non-invasive telemetric monitoring devices—a T_*sk*_ device positioned in a girth belt, an orally administered GI pill (T_*c*_), a global positioning system (GPS), and an HR monitor.

## Materials and Methods

### Horses

Thirteen mainly Arabian (*n* = 10) endurance horses participated in the study: 7 geldings; 6 mares; age, 9.5 ± 2.8 years; body mass (BM), 479 ± 68 kg; body condition scores varied from 2 to 3 out of 5. Two cross-Arabians and one crossbred (quarter horse—thoroughbred) were also involved (Table 1). Coat color included bay (*n* = 3), chestnut (*n* = 6), and gray (*n* = 4), and the color was scored as follows: dark (bay and chestnut, *n* = 9) compared to light (gray) (Table 1). Relevant rider and horse performance history and the Bureau of Meteorology (B.O.M.) (60) station output information were recorded (Table 1; Supplementary Material S1). All the horses were deemed to be fit and healthy based on the veterinary inspection conducted before the competition and following each 40-km loop according to AERA riding rules (50). The horses were sourced on a voluntary basis through the South Australian Endurance Riders Association (S.A.E.R.A.), and all the owners signed a written consent form. The study was approved by the University of Adelaide Animal Ethics Committee (project No. S-2011-224).

**Table 1:** Study population characteristics and monitoring devices.

**Horse number**	**Sex**	**Age (y)**	**Breed**	**Body mass (kg)**	**Coat color**	**Distance (km)**	**GI Pill Y/N**	**GPS/HR Y/N**	**B.O.M. (**°**C) (T_***a***_), min - max**	**Sweating score post-exercise, 1 tot 3**
1	G	11	Arab	669	Gr	80	Y	Y	13–26	2
2	G	9	Arab	484	B	80	Y	Y	13–26	1
3	M	13	Arab	426	C	80	Y	Y	6–19	2
4	M	7	QH x TB	470	C	80	Y	Y	6–19	1
5	M	11	Arab	450	C	80	Y	Y	6–19	1
6	M	8	Arab	370	Gr	80	Y	Y	6–19	2
7	M	9	Arab	450	C	80	Y	∧	3–22	2
8	G	11	Arab	470	Gr	80	Y	Y	3–22	3
9	G	7	QH	490	C	80	–	Y	7–13	3
10	G	10	Arab x TB	484	C	80	–	Y	7–13	3
11	G	5	Arab	458	B	40	–	Y	7–13	1
12	G	7	Arab	525	B	80	–	Y	3–22	1
13	M	15	Arab	480	Gr	80	–	∧∧	3–22	2

*Horses 1-13: 13 endurance horses: G (n = 7), M (n = 6). Arabian, including part-Arabian horses, QH, quarter horse; TB, thoroughbred; x, crossbred; G, gelding; M, mare; Gr, grey; C, chestnut; B, bay. The riders' and horses' performance history includes: age start, indicating age (years) when the horse started competing; horse experience, indicating number of years active in competition (40 km or more); GI pill, gastrointestinal pill; GPS, global positioning system; HR, heart rate monitor (Polar); B.O.M, Bureau of Meteorology; the local station closest to the location of exercise at varying km distances from the actual event (in total, 4 endurance locations, distance ranged from 5.3 to 53 km; -, no; Y, yes; ^∧^indicates HR only; ^∧∧^indicates second 40 km only*.

### Study Design

The horses competed over distances of 40 km (*n* = 1), 80 km (*n* = 10) or 100 km (*n* = 2), with each 40-km loop followed by a 60-min recovery period. The endurance horses exercised at four different locations with altitudes ranging from 4 to 462 meters above sea level. Following each 40-km exercise loop, the sweating response was graded, scoring from 1 to 3 (1: mild wet and white foam areas around head, neck, saddle, and inside hindlimbs, 2: moderate dripping sweat from the body, 3: extensive dripping sweat from the body; Table 1). In addition, the horses were immediately cooled down for average an duration of 10 min by pouring buckets of tap water (estimated average, 20°C) over their bodies and subsequently scraping it off. Following each loop, a recovery period of 60 min was allowed during which inspection of the horses for “fitness to continue” was performed, including checking for the presence of an HR below 60 beats per minute (bpm) by independent endurance veterinarians under the regulations of the Australian Endurance Riding Association (50). Horses were allowed to drink water and eat hay *ad libitum* during the 60-min rest period in a shaded area.

#### Simultaneous Continuous Monitoring of Skin Temperature (T_*SK*_) (°C) and Core GI Temperature (T_*C*_) (°C)

**Figure 1:**
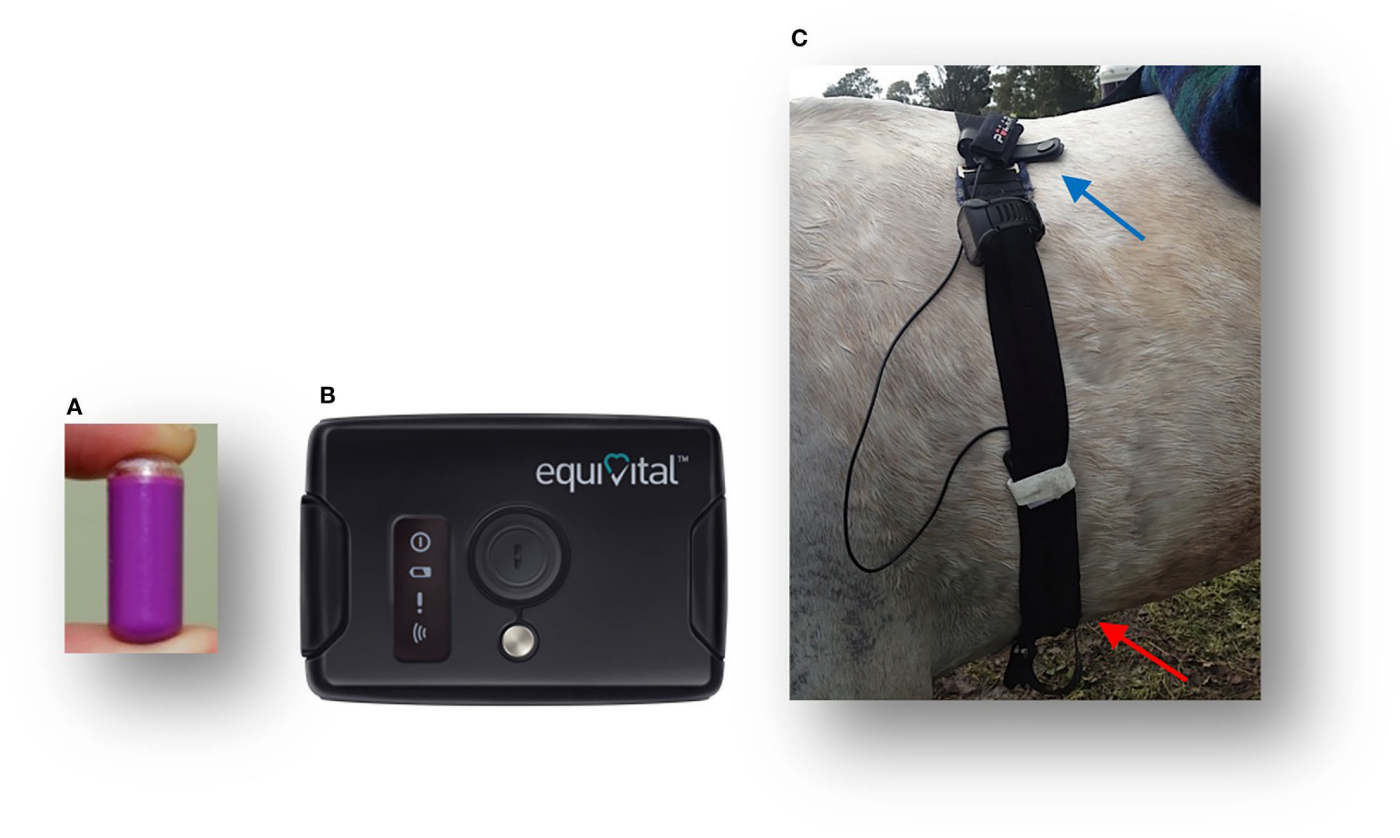
T_*sk*_ and T_*c*_ monitoring equipment: Jonah gastrointestinal temperature pill **(A)**; an external receiver Equivital® Sensor Electronics Module (SEM) with an infrared sensor to measure T_*sk*_ **(B)**; a modified belt for use on horses with the GPS Garmin®Watch and Polar® electrodes (identified by the blue pointer), with the red pointer, indicating the SEM device, including the T_*sk*_ thermistor position **(C)**.

The T_*sk*_ (°C) was continuously recorded using an infrared (IR) sensor measuring 78 × 53 mm located in the Sensor Electronics Module (Figure 1B) (SEM, EQ02 Equivital data Logger®, Hidalgo, UK), with a 0–60°C temperature range, an emissivity of 1 and ± 0.3°C accuracy according to the manufacturer's specifications. The SEM device was located ventrally in a pocket of a modified Equivital Sensor Belt® fitted around the saddle girth (Figure 1C). The GI temperature (T_*c*_) (°C) was continuously telemetrically recorded using the ingestible GI pill (*n* = 8) (Figure 1A) as previously described (T_*c*_ data are to be found in the Supplementary Material S1). The T_*sk*_ and T_*c*_ data were recorded every 15 s and uploaded and processed in the Equivital Software Manager®.

#### Monitoring of Traveled Distance, Speed, and Heart Rate Over Time

For each horse, the distance traveled and speed achieved were recorded telemetrically using GPS monitoring equipment (Garmin Forerunner 910XT GPS Watch® Garmin Ltd., Schaffhausen, Switzerland) attached to the gullet of the saddle (Figure 1C). In addition, the HR was recorded by the Garmin Watch using Polar electrodes (Polar Electro®, Kempele, Finland) (Figure 1C) (61). The GPS and HR data were recorded every second and uploaded from the Garmin watch to the Garmin Connect and processed in the Garmin Training Centre.^1^

#### Ambient Environment

Horses exercised under varying degrees of solar radiation during the Australian winter months (June–August). On each data collection day, the ambient temperature (T_*a*_, °C) and relative humidity (RH, %) were recorded continuously every 30 s in a shaded section of the rest area using a data logger device (OnsetHOBO Pro V2 logger temp/RH U23-00®, Onset Computer Corporation, Bourne, Maine, USA). In addition, T_*a*_ data were obtained from the nearest B.O.M. weather station, presented in Table 1. The estimated wet bulb globe temperature (WBGT) was derived from a WBGT chart (B.O.M).

### Data Processing

Recordings of each exercise period of 40 km and each recovery period following that exercise loop were processed. The net AUC (the baseline set at rest T_*sk*_ and T_*c*_) was calculated using the trapezoidal method of T_*sk*_ (and T_*c*_) over time expressed as °C × minutes. The net AUC was summated to present the cumulative T_*sk*_ – time distribution (62). The net AUC T_*sk*_ provided an estimate of the dynamic thermal response to the thermal load on the skin. This thermal load on the skin during exercise and the recovery included the T_*c*_ and T_*a*_ together with solar radiation.

#### Statistical Analysis

All data are presented as mean ± SD (range). Comparison and correlation analyses were performed using IBM SPSS Statistics 26.0 software or GraphPad Prism version 9.3.0 for MacOS, GraphPad Software, San Diego, California USA^2^. Different approaches were taken to evaluate the potential of the T_*sk*_ data as a reliable proxy to assess the thermoregulatory response. The relationship between T_*sk*_ and T_*c*_ was assessed using scatterplots (8 horses each performing two subsequent 40-km loops). In addition, maximum T_*sk*_ and T_*c*_ and the time to reach maximum T_*sk*_ and T_*c*_ were compared using the paired *t*-test. Delta T_*sk*_ during exercise and recovery periods was compared. The association between T_*sk*_ and T_*c*_ at different points in time and the association with HR or coat color were analyzed using a general linear model ANOVA (when no significant effects of horse identity and treatment interaction were indicated and subsequently removed using backward model selection). Statistical significance was set at α < 0.05.

## Results

All the horses completed their exercise trials without any adverse occurences. The Equivital belt became dislodged in Horse 1 at the end of the first 40-km loop, causing T_*sk*_ and T_*c*_ data loss. As a result, additional modifications were applied to the belt for the subsequent recordings by fitting sturdy straps sandwiched into the belt to stabilize the girth position (Figure 1C). During recovery after the first loop, T_*sk*_ was not recorded in Horses 1 and 2 due to the owners' premature removal of the belt. The sweating response varied from 1 to 3 out of a score of 3 for all the horses (Table 1). The T_*c*_ was recorded in 8 horses over 80 km (previously published, Supplementary Material S1) (2).

### Environmental Field Conditions

The T_*a*_ and RH were successfully recorded between 5.00 a.m. and 3.00 p.m. on all occasions. The T_*a*_ was relatively cool with a mean minimum of 6.7 ±.4°C and mean maximum of 18.4 ± 2.9°C (B.O.M.) (Table 1). More specifically, the T_*a*_ on the four separate days of endurance exercise showed a minimum value of 13.4, 6.3, 2.8, and 6.6°C, respectively, and a maximum value of 26.3, 19.0, 22.0, and 18.8°C, respectively (HOBO data). The minimum RH ranged from 47.1% to 61.7% to a maximum of 84.8–100% value. Overall mean calculated values were 15.3°C (T_*a*_) and 75.6% (RH), respectively, while the approximate WBGT was <20°C. In summary, all the endurance horses competed in a cool environment.

### Speed and Heart Rate Data

All the horses executed their endurance competition at a mean speed of 14.0 ± 1.4 km h^−1^ over the first 40 km (*n* = 11) and 14.2 ± 2.1 km h^−1^ over the second 40-km (*n* = 11) loop, with a mean HR of 114 ± 13 bpm. An overview of recorded speeds and HR data for individual horses can be found in Supplementary Material S1.

### Individual T_*sk*_ and T_*c*_ Recordings During Endurance Exercise Over Time

**Figure 2:**
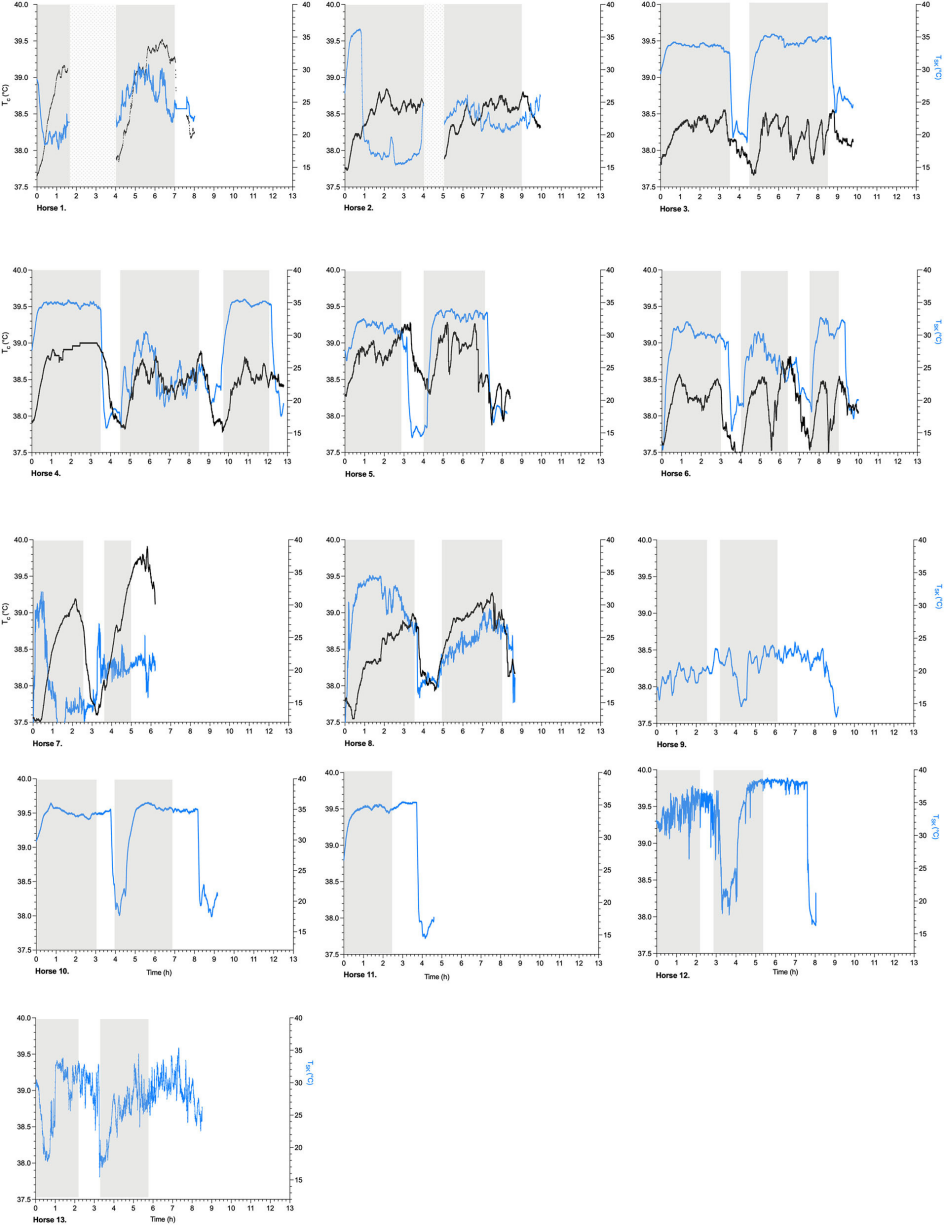
Scatterplots of T_*sk*_, skin temperature (°C, identified as blue) and T_*c*_, gastrointestinal temperature (°C, identified as black) (left y-axis) per subsequent exercise loop of 40 km (gray blocks) (h, hours, x-axis) in endurance horses, Horses 1–13; dotted blocks identify blocks of no data recording; after each exercise loop, the horses were cooled down by pouring buckets of water over their bodies, followed by scraping the water off the bodies for a period of ~10 min.

An overview of the simultaneously recorded individual T_*sk*_ (°C) and T_*c*_ (°C) time profiles is provided for all the horses in Figure 2. All individual T_*sk*_ parameters, their respective descriptive analysis, and specific T_*sk*_ points in time during the 40-km endurance loops are presented in Table 2.

**Table 2:** T_*sk*_ (°C) parameters during 40-km exercise (or extra 20 km in 2 horses exercising over a total of 100 km) and recovery.

**Horse**	**Distance 40 km – 1^**st**^ or 2^**nd**^**	**Net AUC T_***sk***_ (**°**C x min) exercise 40 km**	**Net AUC T_**sk**_ (**°**C x min) recovery 40 km**	**Base T_***sk***_ (**°**C) at start of exercise**	**Mean ±SD T_***sk***_ (**°**C) exercise**	**Min - max T_***sk***_ (**°**C) 40 km exercise**	**Delta T_***sk***_ (**°**C) exercise**	**T_***sk***_ (**°**C) at start of recovery (end exercise)**	**Delta T_***sk***_ (**°**C) first 10 min recovery**	**Delta T_***sk***_ (**°**C) recovery**	**Delta T_***sk***_ (**°**C) end exercise to end recovery/min**	**Mean ±SD T_***sk***_ (**°**C) recovery**	**Min –max T_***sk***_ (**°**C) recovery**	**Time to max T_***sk***_ exercise (min)**	**Time to min T_***sk***_ in recovery (min)**	**T_***sk***_ (**°**C) at end of recovery**	**T_***sk***_ (**°**C) at end of recovery**
1	1st	−743.7	–	28.5	20.7 ± 2.4	17.8–28.4	10.6	21.9	–	–	–	–	–	0	–	23.2	23.2
1	2nd	587.3	−0.78	23.2	26.4 ± 2.6	21.2–31	10.8	24	−0.3	3.7	0.06	23.7 ± 1.1	22.1–25.8	74	21	26.4	22.8
2	1st	−1,328.6	–	26.4	20.9 ± 7.2	15.3–36.2	20.9	24.2	−1	6.6	–	23.3 ± 1.7	–	45	–	24.7	24.3
2	2nd	−515.3	54.9	24.3	22.6 ± 1.4	20.4–26.1	5.7	22.3	−1	5.1	0.09	23.3 ± 1.3	21–26.1	76	20	26	25.9
3	1st	838.1	−624.6	29.4	33.5 ± 0.8	29.4–34.2	4.8	32.5	−9.3	13.9	0.23	21.8 ± 3.5	18.8–32.7	39	29	22.1	22.5
3	2nd	2,820.5	−597.3	22.5	33.9 ± 2.0	22.5–35.4	12.9	34.8	−6.4	11.5	0.19	26.4 ± 2.7	23.4–34.9	72	23	24.6	24.4
4	1st	1,382.4	−85.5	27.7	21.0 ± 1.3	27.8–35.4	7.7	34.2	−12.3	18.4	0.31	21.8 ± 3.5	18.8–32.7	110	28	18.2	18.2
4	2nd	1,422.9	169.2	18.2	24.0 ± 3.2	15.8–30.5	14.7	24.7	−0.6	7.5	0.13	22.1 ± 1.8	19.3–26.5	79	16	26.8	26.8
4	*Extra 20*	*(1,051.1)*	*(*–*468.1)*	*26.9*	*34.4 ± 1.6*	*26.9*–*35.5*	*8.5*	*34.7*	–*8.6*	*17.2*	*0.29*	*22.8 ± 4.7*	*17.6*–*34.7*	–	*27*	*19.5*	*19.6*
5	1st	570.8	−635.5	27.6	30.7 ± 1.5	26.1–32.5	6.4	28.3	−10	15.4	0.26	16.7 ± 3.5	14.3–29.7	56	26	16.2	16.1
5	2nd	2965.8	−997.3	16.1	31.9 ± 3.8	15.8–34	18.2	33.5	−12.9	16.9	0.28	19.2 ± 3.3	16.6–33.4	79	16	18.2	18.2
6	1st	2,736.5	−408.7	13.7	29.0 ± 4.0	12.4–32	19.6	29.7	−0.8	14.5	0.24	22.8 ± 5.3	15.3–29.5	74	12	19.3	19.5
6	2nd	995.4	251.0	19.5	26.2 ± 2.8	19–30.5	11.5	24.6	0.8	6.5	0.11	23.2 ± 2.2	20.1–26.6	55	19	20.1	20.1
6	*Extra 20*	*(809.8)*	*(*–*427.0)*	*20.1*	*29.0 ± 4.1*	*18.1*–*32.7*	*14.5*	*31*	*1.2*	*15.1*	*0.25*	*23.8 ± 5.9*	*17.2*–*32.3*	*36*	*46*	*20.1*	*20.1*
7	1st	960.6	−3	11	17.7 ± 6.3	10–32	22	13.3	−0.7	15	0.25	16.4 ± 3.9	12.1–27.1	26	10	18.9	19.2
7	2nd	80.4	97.5	19.2	20.4 ± 1.0	18.2–23.9	5.7	19.7	0.8	9.5	0.16	20.8 ± 1.2	15.8–25.3	64	18	20.2	20.2
8	1st	4,170.6	−547.4	10	30.4 ± 4.1	10–34.5	24.5	25.9	1	11.4	0.19	19.3 ± 3.1	15.8–34.5	75	10	20.3	20.2
8	2nd	884.2	110.8	20.2	24.8 ± 2.1	19.2–29.4	10.2	26.7	−1	12.2	0.20	23.8 ± 3.1	15–27.2	150	19	18.3	18.8
9	1st	535.8	133.2	18.8	19.8 ± 1.7	15.6–23.5	7.8	23.4	−2.8	8.9	0.15	18.0 ± 2.6	14.6–23.4	179	16	22.6	22.6
9	2nd	−68.3	−172.4	22.6	22.3 ± 0.9	18.7–24.4	4.7	23.4	−3.2	10.4	0.17	17.6 ± 2.8	13–32.3	141	20	14.3	14.5
10	1st	934	−628.4	29.8	34.2 ± 1.1	29.9–36	6	34.7	0.3	17	0.28	23.9 ± 5.9	17.7–35	44	30	26.1	26.2
10	2nd	1,825.3	−852.4	26.2	34.7 ± 1.5	26.2–36.1	10	34.8	−15.3	17	0.28	20.4 ± 2.3	17.5–32.3	63	26	21.2	20.9
11	1st	1,691.6	−944.0	26.5	34.3 ± 1.4	26.6–35.5	9	35.4	−15.9	21	0.35	18.5 ± 5.7	14.5–35.4	179	27	17.7	17.7
12	1st	4,748.6	−597.2	32.3	30.9 ± 7.6	9.5–37.5	29	35.3	−10.4	19	0.32	22.8 ± 3.5	17.9–35	152	26	28.4	28.7
12	2nd	1,356.4	−524.7	28.7	37.4 ± 1.7	28.7–38.7	10	38.1	−19.4	22.2	0.37	19.6 ± 4.9	16.3–38.2	90	29	16.8	16.8
13	1st	−204.5	−169.3	29.5	24.2 ± 4.4	17.9–33.8	16	25.5	4.6	17	0.28	22.6 ± 4.6	15.5–32.8	84	18	26.8	26.4
13	2nd	453.9	−45.1	26.4	28.6 ± 2.5	21.6–35.2	14	27.9	2.6	11	0.18	27.1 ± 2.2	22.6–33.4	196	22	24.1	26.2

*Data are presented as mean ± SD. T_sk_, skin temperature; AUC, area under the curve; min, minutes; min-max, minimum to maximum; italic indicates an extra loop of 20-km exercise (total, 100 km, n = 2 horses); delta (°C change), T_sk_ change during exercise, and recovery periods, including the first 10-min recovery period; – indicates no data collected/not available*.

### Overall T_*sk*_ Profiles and Comparison to T_*c*_

The overall T_*sk*_ profiles during endurance exercise and recovery and their associated parameters are presented in Table 3, showing a mean time to maximum T_*sk*_ of 88 ± 51 min (*n* = 13). The mean maximum T_*sk*_ during exercise was 32.4 ± 4.3°C, and the mean minimum T_*sk*_ during recovery was 17.3 ± 3.1°C (*n* = 13). The mean overall response of T_*sk*_ was 1,164 ± 1,448°C × minutes for each 40-km exercise period. During recovery, the T_*sk*_ response was −305 ± 388°C × minutes (Table 3). The T_*sk*_ and T_*c*_ profiles over time were compared in the 8 horses, and no relationship was found (Figure 2).

**Table 3:** Overall T_*sk*_ and T_*c*_ variables during exercise and recovery of endurance exercise in a cool environment.

**Variables**	**Endurance 40 km *n* = 13** (*n* = 8^*^)
Duration (minutes) exercise	198 ± 63
Duration (minutes) recovery	60
T_*sk*_ (°C) overall	27.8 ± 5.6 (17.71–37.37)
Base T_*sk*_ (°C) (at start-of-exercise)	23.1 ± 6.1 (10–32.3)
Min T_*sk*_ (°C) exercise	19.8 ± 6.2 (9.5–29.9)
Max T_*sk*_ (°C) exercise	32.3 ± 4.3 (23.5–38.7); 31.6 ± 3.5°C^*^
T_*sk*_ (°C) exercise	27.2 ± 5.7 (17.7–37.4)
Time to max T_*sk*_ exercise (minutes)^*^	88 ± 51 (0–196); 67 ± 34^*^
Delta T_*sk*_ (°C) exercise	12.5 ± 6.6 (4.7–29); 12.9 ± 6.4^*^
Net AUC T_*sk*_ exercise (°C × minutes)	1,164 ± 1,448 (−1,329 to 4,749); 1,114 ± 1,469^*^
T_*sk*_ (°C) at end-of-exercise	28.0 ± 6.1 (13.3–38.1)
Min T_*sk*_ (°C) recovery	17.3 ± 3.1 (12.1–23.4); 17.7 ± 3.3^*^
Max T_*sk*_ (°C) recovery	30.9 ± 4.1 (23.4–38.2)
T_*sk*_ (°C) recovery	21.5 ± 2.8 (16.4–27.1
Delta T_*sk*_ (°C) recovery	13.0 ± 5.1 (3.7–22.2)
Delta T_*sk*_ (°C) first 10 min recovery	−4.7 ± 6.7 (−19.4 to 4.6)
Delta T_*sk*_ first 10 min recovery/minute (°C/min)	−0.5 ± 0.7 (−1.9 to 0.5)
Number of horses T_*sk*_ > 39°C^**^	None
T_*sk*_ (°C) at end-of-recovery 40 km (*n* = 25)	21.8 ± 3.8 (14.5–28.7)
Number 40 km periods T_*sk*_ returned to base T_*sk*_ at the end of 60 min recovery	14/25
Net AUC T_*sk*_ recovery (°C × minutes)	−305 ± 388 (−997 to 251); −230 ± 392^*^

*Data are presented as overall mean ± SD. T_sk_, skin temperature; AUC, area under the curve; T_c_, GI pill temperature; max T_c_ or T_sk_, maximum T_c_ or T_sk_; n, number identified only if different 40-km exercise periods*.

* *Indicates a total of 8 horses (comparison T_sk_ to T_c_ in 8 horses; total, 16-x-40-km periods)*;

** *T_sk_ > than 39°C based on (21)*.

**Figure 3:**
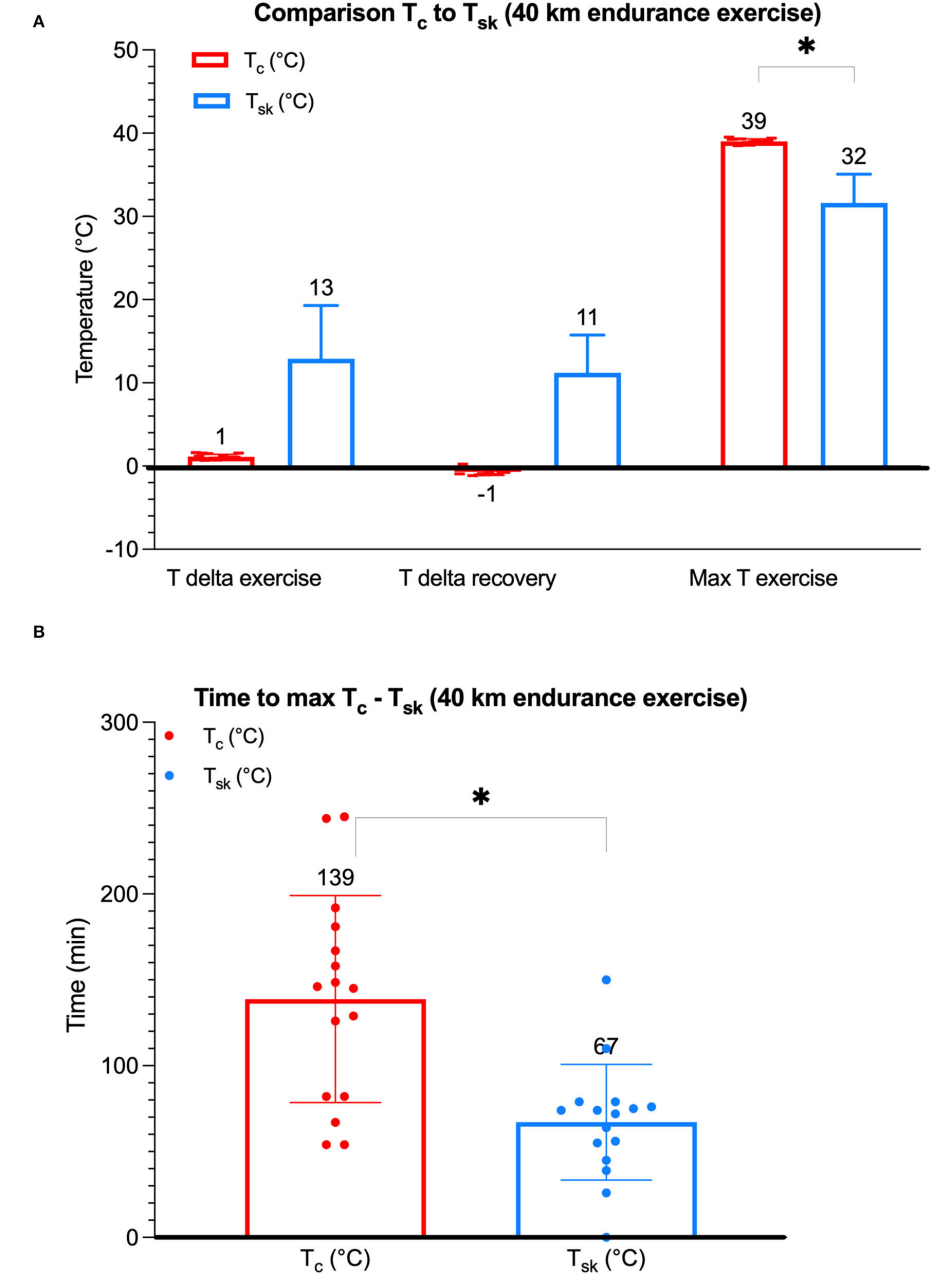
A boxplot diagram depicting T_*c*_ and T_*sk*_, respectively, in endurance horses. Mean (a horizontal line) ± SD (error bars) of individual maximum T_*c*_ (°C, *n* = 8) and maximum T_*sk*_ (°C, *n* = 8) values. There was no significant association between T_*sk*_ and T_*c*_; no significant difference between delta T_*sk*_ exercise and recovery (*p* = 0.41); a significantly greater maximum T_*c*_ when compared to maximum T_*sk*_ (*p* = 0.0002) **(A)**; and a significantly increased time to maximum T_*c*_ than T_*sk*_ (*p* = 0.0004) **(B)**; *indicates a significant difference.

Different T_*sk*_ and T_*c*_ points in time were compared to assess associations. Interestingly, the only significant correlation found was between the T_*sk*_ (°C) at the end-of-exercise period and the T_*c*_ (°C) at the end-of-recovery period (*F*_1,14_ = 5.519, *p* = 0.034). More precisely, a higher T_*sk*_ at the end-of-exercise period was associated with a lower T_*c*_ at the end-of-recovery period. The additional analyses revealed no significant correlations between T_*sk*_ (°C) and T_*c*_ (°C), including no correlation between T_*sk*_ at the start-of-exercise period (baseline T_*sk*_) and the maximum T_*c*_ (*F*_1,14_ = 0.127, *p* = 0.727). The study could not identify a significant effect of time to maximum T_*sk*_ (67 min) during exercise on the maximum T_*c*_ (39°C) (*F*_1,14_ = 0.001, *p* = 0.978, *n* = 8). On all occasions, peak T_*c*_ values (39°C) were significantly greater than peak T_*sk*_ values (32°C) (*p* = 0.0002) (Figure 3A). In addition, in all cases, there was a significantly shorter time to maximum T_*sk*_ (88 min) compared to the time to maximum T_*c*_ (139 min) (*p* = 0.0004) (Figure 3B).

Delta T_*sk*_ data were compared, and, when comparing exercise and recovery periods, no significant difference could be found between the two delta T_*sk*_ (°C) (mean 13°C and 11°C, respectively) (*p* = 0.41) (Figure 3A). The delta T_*sk*_ was greater when compared to delta T_*c*_ on all occasions (Figure 3A). Also, delta T_*sk*_ and T_*c*_ points in time were compared to assess associations. A correlation between the delta T_*sk*_ during cooling in the first 10 min of the recovery period and the T_*c*_ at the end of the recovery period was not identified (*F*_1,13_ = 1.348, *p* = 0.266).

Additionally, there were no significant effects of coat color on the maximum T_*c*_ (*F*_1,14_ = 0.010, *p* = 0.920) nor on the maximum T_*sk*_ (*F*_1,22_ = 0.015, *p* = 0.904). Furthermore, coat color was not associated either with delta T_*sk*_ during exercise (12.5 ± 6.6) (*F*_1,22_ = 1.098, *p* = 0.306) or delta T_*sk*_ during recovery (13.0 ± 5.1) (F_1,21_ = 1.246, *p* = 0.277).

### Evaluation of Heart Rate in Relation to T_*sk*_

Additional analysis to evaluate the relationship between the T_*sk*_ and HR < 60 bpm revealed no significant correlation between the T_*sk*_ at the end of exercise and the duration to HR < 60 bpm (*F*_1,13_ = 4.019, *p* = 0.066). In conclusion, the study did not identify an association between HR recovery and the recorded T_*sk*_ during endurance exercise and recovery.

## Discussion

This is the first study to simultaneously monitor both T_*sk*_ and T_*c*_ continuously by means of several telemetric temperature recording devices on exercising horses in the field. The thermal sensors functioned correctly throughout the study, hence, there was little to no data loss. Consistent with our previous field study (2), the current study confirmed a substantial inter-individual variability in the T_*sk*_ time profiles despite execution of the same exercise protocol. Similar findings have been reported in human athlete studies and underline the physiological complexity of the mammalian thermoregulatory response, which is governed by a plethora of individually intrinsic variables (63–66).

With respect to T_*sk*_ and T_*c*_ monitoring in the current study, there was a lack of correlation between continuous monitoring of T_*c*_ and T_*sk*_. Nevertheless, the association of a higher single point T_*sk*_ at the end-of-exercise period with a lower T_*c*_ at the end-of-recovery period is an interesting finding in the current study.

Up until now, a limited number of studies, almost all of which were treadmill based, monitored the T_*sk*_ continuously in the horses during the exercise and compared the T_*sk*_ to T_*c*_ (38–40, 42, 52). Only one recent field study has been performed, monitoring T_*sk*_ and T_*re*_ (20). The treadmill studies confirmed the lack of correlation between T_*sk*_ and T_*c*_. For example, two submaximal exercise studies using arterial blood temperature compared the effect of different environments on thorax surface T_*sk*_ and showed T_*sk*_ was different from T_*c*_ (38, 42). Two high-intensity studies reported tail surface T_*sk*_ responses to cooling methods and acclimation, respectively, and both studies showed T_*sk*_ recordings were different from the T_*c*_ recordings (40, 41). Apart from those studies, only one laboratory-based high-intensity exercise study, comparing exercise in four horses in a cool vs. hot environment, suggested that the tail T_*sk*_ evolvement pattern seemed to follow the T_*c*_ pattern (using arterial blood temperature), although a statistical correlation was not investigated (39). A recent laboratory equine exercise study using the implantation of microchips, measuring muscle temperature (defined as “outer shell temperature” in that study) which may be extrapolated to field exercise in the future (67). There was a good correlation between central venous temperature (CVT) evolvement and outer shell temperature during a short bout of exercise (8–11.5 min) until CVT reached 41°C, although the outer shell temperature was reported to lag behind CVT during the recovery phase. While most of these experimental studies did not reveal a statistical correlation between T_*sk*_ and T_*c*_, efforts to further investigate T_*sk*_ continue as the technique could easily be employed in the field. Consequently, wearable thermo-sensor techniques are being upgraded at an ever-increasing pace.

### Continuous T_*sk*_ Sensor Recording During Field Exercise

Ongoing efforts to identify a reliable proxy for continuously monitoring the thermoregulatory response in horses during field exercise are not always successful or practical. A more invasive method with thermistors placed in blood and brain was used in three horses during free field exercise and reported a good correlation; however, for obvious reasons, this is not easily applicable in practice (51). Several field studies have investigated less-invasive continuously monitoring approaches, such as the intra-uterine temperature (two mares) or the GI temperature; both approaches (intra-uterine and GI) showed a good correlation with the T_*c*_ (18, 19, 68). A recent study has continuously monitored surface T_*sk*_ using i-Button® and simultaneous T_*re*_ during two canter bouts of 4.5 min of field exercise at a speed ranging from 6.7 to 7.5 meters per second (20). The T_*sk*_ was continuously monitored at the level of the rump and shoulder regions in 10 eventing horses using a cross-over study design. The study showed that pre-exercise cooling resulted in a delta T_*sk*_ ranging from −2.3 to −3.3°C and a reduced median T_*re*_ of 0.3°C, which peaked at 9 min into recovery, compared to the control group (20). Although the study did not investigate correlations between T_*sk*_ and T_*c*_, the effect of lower T_*sk*_ pre-exercise on a reduced T_*re*_ is of interest, and consistent with previous human sports studies (69, 70).

In brief, the current reliance on continuous T_*sk*_ sensor recordings during field exercise has been proven to be inconsistent and unreliable as a proxy for the thermoregulatory response. This is consistent with a study comparing the effects of precooling in 10 human athletes to estimate the T_*c*_ (using a GI pill) (71).

### Comparing T_*sk*_ With T_*c*_

When comparing the delta T_*c*_ with delta T_*sk*_ in the current study, the delta T_*sk*_ was greater during endurance exercise as depicted in Figure 3A. However, a laboratory-based high-intensity equine exercise study using thermocouples attached to the skin with tape and located in pulmonary artery blood to continuously monitor T_*sk*_ and T_*c*_ in six horses revealed a delta T_*sk*_ of 2.5°C (42) similar to the delta T_*c*_ value in the current study (3°C). Associations between T_*sk*_ and T_*c*_ were not evaluated in that study; however, the difference in the exercise duration may indicate a difference in thermoregulatory activity over time, namely, 200 min of endurance exercise in the current study vs. average of 40 min in the former study evidenced by the end-of-exercise T_*c*_ > 41°C (42).

Another interesting finding was the significantly higher time to maximum T_*c*_ when compared to the time to maximum T_*sk*_ (Figure 3B)_._ This finding suggests that the endurance horses in the current study performing in a cool environment were efficiently thermoregulating during exercise without the development of hyperthermia (T_*c*_ > 39°C) as has been documented previously (2). On the other hand, a short duration of high-intensity exercise in more challenging environmental conditions may trigger T_*c*_ > 41°C and, consequently, requires dissipation of excess MH to occur post-exercise (38, 42, 53).

Despite all these ongoing efforts to practically incorporate surface T_*sk*_ monitoring into thermoregulation and wellbeing monitoring in the field, researchers should always keep in mind the possible factors that challenge a potential correlation between T_*sk*_ and T_*c*_. In addition, the monitoring device used must be able to correctly function and cope with the practical conditions under which horses exercise and compete. Important factors are environmental variables; among which are weather conditions, and whether or not additional cooling is applied. Furthermore, the type of temperature sensor equipment and the anatomical site at which the T_*sk*_ equipment is placed have their influence, together with individually intrinsic horse-related factors.

### Environmental Factors

#### Factors Influencing T_*sk*_ and T_*c*_ Evolvement and How They Relate to Each Other

The T_*sk*_ at any site on the skin surface reflects a balance between heat being delivered to the skin by arterial blood, body, and local skin metabolism, and heat exchange with the environment by convection, radiation, and evaporation. Any factors that interfere with this balance can change the T_*sk*_. Many factors that modulate T_*c*_ evolvement during exercise simultaneously influence T_*sk*_, such as a plethora of performance capacity parameters, as well as environmental conditions. Environmental factors can easily and quickly change T_*sk*_ without directly affecting T_*c*_ (1, 47, 59, 72–74). These factors include the T_*a*_, solar radiation, soil radiation, humidity, shade, and wind speed (air movement). For example, a varying T_*a*_ ranging from 20 to 30°C was directly related to the onset of skin vasodilation and sweat evaporation (72), while, on the other hand, a low T_*a*_ was shown to induce a lower sensitivity (50%) of percutaneous T_*sk*_ microchips in 52 foals and 30 adult horses to identify fever compared to measuring T_*sk*_ in a hotter T_*a*_ (29°C) (73). That would mean that, in case of fever (also known as an increased T_*c*_ set-point), a cool sunless environment renders T_*sk*_ monitoring using microchips less representative for T_*c*_ monitoring. In addition, Holcomb et al. (74) demonstrated that T_*sk*_ and T_*re*_ were highest at the peak solar radiation during the day. The T_*sk*_ sensor in the current study was located ventrally on the chest of the horse covered by the belt and the girth, thus avoiding T_*a*_ effects, such as solar radiation.

It is common practice to cool down endurance horses during the recovery period between subsequent exercise loops. Cooling down was also applied in the current study design in a real-life competition context. The goal was to challenge the temperature monitoring devices with real circumstances in which they would be required to function. With respect to cooling down approaches, the mechanism by which the thermoregulatory systems are challenged greatly depends on how the loss of heat counteracts MH production through non-evaporative pathways as well as evaporative methods. The evaporative exchange of heat of the skin with the environment depends on the thermal gradient between T_*sk*_ by local skin perfusion and its immediate environment, including vapor pressure, airflow, and solar radiation, especially during field exercise (17, 49, 75). At the end-of-exercise period, the cooling of sport horses is standard, especially in endurance and 3-day eventing competitions. However, post-race cooling methods are not standardized in the racing industry. The duration of cooling endurance horses in the field is, on average, 10 min based on each owner's judgment, which could include HR monitoring. The mean end-of-exercise T_*sk*_ in our study with endurance horses exercising in a cool environment (mean, 15.3°C) was 28°C, and no horses developed a T_*sk*_ higher than 39°C.

On the other hand, exercise studies in warmer environments documented a post-exercise T_*sk*_ higher than 39°C. For example, a recent report has revealed that 28 out of 38 horses exercising in a hot, dry environment (mean T_a_, 38.8°C), and 6 out of 37 horses exercising in a warm, humid environment (mean T_a_, 31.1°C) showed a post-exercise IRT T_*sk*_ higher than 39°C. These researchers suggested horses recording T_*sk*_ higher than 39°C were at risk of developing heat stress and EHI and used this T_*sk*_ response as an indicator for racehorses requiring cooling (21). The association between T_*sk*_ and EHI risk could be physiologically explained by a low T_*c*_-to-T_*sk*_ gradient, therefore decreased capability to transfer MH to the skin, thus compromising the dissipation of MH by evaporation (21, 56).

A similar mean IRT T_*sk*_ of 40°C was recorded at the end of exercise in a recent study, evaluating cooling methods in racehorses in a warm environment (mean T_a_, 31.8°C) (35). A T_*sk*_ higher than 39°C is consistent with earlier laboratory-based studies in a warm T_*a*_ (29.1°C and 31.1°C, respectively) (40, 45). In retrospect, scraping off water from the horses during cooling down was not the most optimal approach since Takahashi et al. (35) favored continuous application of cold water without subsequently scraping it off.

### T_*sk*_ Equipment-Related Features and Location

#### Equipment to Measure T_*sk*_

Within the rapidly expanding wearable digital device industry, surface T_*sk*_ monitoring devices are constantly being upgraded to provide data output. However, in that respect, the critical question remains: how should we interpret those data? Overall, three different types of temperature sensor equipment are reported: thermistors (such as microchips), thermocouples, and IRT devices, with IRT being the most studied device recently in horses (21, 22, 26, 32–37, 76–80). It is essential to understand that those sensors use different physical processes to obtain data, which may result in significant differences in data output. These sensor surface T_*sk*_ differences due to the type of equipment may show only a minor bias, which may prove to be clinically meaningful (75). For example, a study comparing IRT and thermocouples at single pre-exercise, intra-exercise and post-exercise points in 12 human athletes revealed a poor Bland Altman agreement and low reliability between the different methods (81).

To produce IRT imaging to picture surface T_*sk*_ of different parts or the whole body, a remote IRT camera positioned at 30-cm proximity to the skin surface has been recently evaluated with varying results (26, 37, 82). For example, a study compared IRT T_*sk*_ to T_*re*_ in 40 adult horses and concluded T_*sk*_ was not an accurate method to determine the T_*c*_ (82). The remote position of the IRT held far from the skin has the advantage of not interfering with the local T_*sk*_ balance, although the remote T_*sk*_ measurement will be partly affected by the adjacent environment surrounding the skin (22, 23, 75). The temperature sensors that were used in the current study were in direct contact with the skin and covered by a belt. This belt might have interfered with the local thermal conductivity and the local evaporative cooling capacity and thus might have delayed equilibration of the local T_*sk*_ with the surrounding skin. On the other hand, an adequate and essential sensor-to-skin contact was ensured by the position of the sensor in the belt. Furthermore, the skin surface covered by the sensor was small enough to prevent causing local skin changes (22, 75).

We were unable to calibrate and validate the IR sensor prior to the study; however, studies of different T_*sk*_ recording methods and comparisons with a certified thermocouple in a thermo-statically controlled water bath are extremely rare. One study evaluated sensor systems in human athletes during rest and exercise in a hot environment and revealed a good agreement for employing a telemetric thermistor system when compared to the standard hard-wired thermistor system and a poor agreement for using a thermal camera (83).

In summary, IRT techniques differ widely in human and equine medicine, including positioning of the camera and environmental control measures (22, 24, 75). A consensus guideline has been developed only recently, addressing the multiple data collection methods of the human T_*sk*_ using IRT (84), while Soroko and Howell (22) described a protocol using IRT in equine medicine.

### T_*sk*_ Equipment Location

The anatomical location of the sensor on the horse to record T_*sk*_ measurements has been shown to influence T_*sk*_ results (22, 23, 26, 29, 37, 47). For example, remote IRT was used to evaluate differences between 10 locations on the body during two seasons in the year with the highest T_*sk*_ recorded at the level of the chest (22.5°C) and shoulders (20.4°C) in horses at rest in a cool T_*a*_ (mean, 6.7°C) (26). In another study, the IRT T_*sk*_ was greatest at the shoulder area when compared to three other T_*sk*_ locations measured at the start and the end of 20-min exercise (32.3°C and 34.2°C, respectively) in a moderate T_*a*_ (mean, 23°C) (37). The results of our study in a cool T_*a*_ (mean, 15.3°C) revealed a mean T_*sk*_ measured at the lower chest area of 23.1°C during an average of 200-min exercise and a mean T_*sk*_ of 21.8°C during recovery. The different T_*sk*_ values between the current study and Soroko et al. (37) illustrate the effect of exercise intensity (submaximal vs. maximal) and duration (long vs. short). The differences in T_*sk*_ over several body areas may relate to the varying networks of blood vessels in those body regions and their vasodilation to exchange thermal heat with the proximal environment (23, 55, 56, 59). It is essential to note that both monitoring methods share some vasomotor or endocrine mechanisms, although they present differences depending on the degree of heat dissipation or retention that the organism needs. Consequently, monitoring of T_*sk*_ in the current study revealed a physiological response of the local T_*sk*_ to the changes of T_*c*_ during endurance exercise over time, although the responses were not correlated.

While IRT cameras are increasingly used in equine sports medicine, this method involves a single point in time measurement. One exception is the study by Soroko et al. (37), who reported dynamic IRT monitoring every 15 s during treadmill exercise. To be precise, a review of the use of IRT in human endurance athletes reported that 25 of the 45 studies were conducted over the last 5 years (2017–2021), but, up until now, only five real-life field endurance studies have been performed (24). The latter review concluded that further analysis is required to assess whether T_*sk*_ could be used as a reliable proxy to describe real-time thermoregulation (24). Another important relevant finding is that surface T_*sk*_ may be low in human athletes with EHI and, hence, provide misleading information (85). A different IRT method approach is measuring eye surface temperature; that study revealed no relationship with T_*c*_ in horses (86).

### Individual Horse-Related Factors

Horse-related factors include breed, body condition score, age, character (such as nervousness), and skin-related properties, such as sweat rate, skin thickness, blood vessel density, hair coat properties, clipping, and coat color (23, 27, 30, 36, 55, 77, 78, 80, 87). The sweat loss in the current study was subjectively scored from 1 to 3 by E-L.V., and, in retrospect, more accurate sweating scoring based on objective specific phenotypic descriptions would have been a better approach (88). The effect of breed on T_*sk*_ relates to the ratio of BM to the body surface area—the higher the body surface area in relation to the BM, the higher the heat dissipation (72). The low-surface-area-to-mass ratio of the horse results in greater demands being imposed on the thermoregulatory system during long-term submaximal exercise (30, 49, 53). Our study included mainly Arabian horses, known to have a lower BM and, hence, a higher-surface-area-to-mass ratio.

The hair coat length in the current study was similar (all clipped), which is essential as clipping the winter coat resulted in improved heat dissipation during and after exercise, resulting in decreased T_*sk*_ and T_*re*_, as reported in previous studies (27, 30). One of those previous studies used a thermistor probe to evaluate the effect of coat clipping in three horses on both the surface T_*sk*_ and T_*re*_. That study reported no effect of clipping on post-exercise T_sk_, while T_*re*_ was ~0.2°C higher in unclipped horses (30). Indeed, a longer haircoat length limited the thermal imaging in a study assessing T_*sk*_ in mares (77). Furthermore, coat colors may be relevant (87); however, the current study of 13 horses revealed that light or dark coat color had no significant effect on T_*sk*_, which is consistent with a previous study (73). Individual horse-related character differences may exist, such as nervousness that triggers sympathetic nerve activity associated with vasoconstriction of skin blood vessels. This neurophysiological response may explain varying reduced local T_sks_, decreased heat loss, and hyperthermia (23, 55, 78, 89).

### Modeling Using T_*sk*_

While, generally, T_*sk*_ can be easily monitored, the T_*sk*_ in the current study did not provide data suitable for extrapolating to similar changes in the T_*c*_. Consequently, the development of integrative models using T_*sk*_ to determine the heat balance during exercise has been investigated in human studies and in one equine study (87, 90, 91). However, no regression model could predict physiological stress load using single-point IRT T_*sk*_ in 17 human marathon runners in the field (92). A recent approach in human exercise research has investigated the application of models and algorithms using data and variables, such as HR and HR variability, to successfully estimate T_*c*_ (47, 90, 93–95). Physiologically, HR reflects the blood flow rate to the muscles (MH production) and blood flow to the skin (heat loss). For example, recent studies have concluded that combining continuous insulated T_*sk*_ and HR monitoring in 13 and 8 human athletes in a hot (35°C) and warm (25°C) environment, respectively, could provide a predictive model of T_*re*_ or T_*c*_ (using GI pills) (90, 94). In contrast in the current study, HR recovery in the endurance horses was not directly related to T_*sk*_. Further investigation is required into the potential association of T_*sk*_ and HR for accurate predictive modeling of T_*c*_ in equine athletes.

### Association Between Single-Point T_*sk*_ at the End-of-Exercise Period Compared With the T_*c*_ at the End-of-Recovery Period

An interesting finding of the current study performed with the endurance horses was the association of a greater T_*sk*_ at the end of exercise with a significantly lesser T_*c*_ at the end of recovery (60 min). Several theories could be considered to explain this association between T_*sk*_ and T_*c*_: firstly, the raised T_*sk*_ indicates the launch of an active thermoregulatory response to anticipate the increased T_c_, and, once the MH is successfully dissipated, the T_*c*_ decreases. This argument can be coupled with the effect of cooling post-exercise, which may be more prominent when T_*sk*_ is greater and, ultimately, results in higher dissipation of MH and a reduced T_*c*_. Several other field exercise studies in horses have investigated correlations between single-point T_*sk*_ and other variables (21, 32). For example, a recent equine study involving 8 endurance horses has investigated the association between endurance training intensity (1 h at 19 km/h vs. 2 h at 16 km/h vs. 3 h at 20 km/h) and T_*sk*_ using an IRT camera measured at different locations and at different time points. The study identified that the T_*sk*_ at the coronary band increased with training intensity unlike the maximum T_*sk*_ (32).

Aside from the variance in hot vs. cool T_*a*_ in these studies the differences in exercise intensity could explain the dissimilarity between the racehorse study results of Brownlow and Mizzi (5) and the current study involving endurance horses. For racehorses undertaking high-intensity, short-duration exercise, the dissipation of MH occurs post-exercise as opposed to endurance horses, which manage their MH throughout their submaximal long-duration exercise (2, 5, 21, 56). For example, the T_*sk*_ and its evolvement pattern can be related to acute blood flow variances associated with a different type of exercise intensity (25, 95). Overall, in our study monitoring endurance horses conducting exercise during cooler months, the mean T_*sk*_ at the end of exercise was 28°C, while none of these horses had a T_*sk*_ higher than 39°C.

The end-of-recovery period T_*sk*_ showed a considerable individual variation (range, 14.5–28.7°C) despite the application of a uniform cooling protocol. The T_*sk*_ during the 60-min recovery period revealed that the T_*sk*_ returned to the baseline only in over 50% of the 40-km recovery periods. This is in contrast to other studies, which found that after 20 min of treadmill exercise in a hot (32–34°C) and dry T_*a*_ condition, all T_*sk*_ values returned to baseline T_*sk*_ after 60 min, and, after 45 min in a T_*a*_ of 20°C (without cooling), respectively (28, 42). The main difference between the current study and other laboratory-based studies was the continuous T_*sk*_ monitoring during a field exercise in an uncontrolled T_*a*_.

### Limitations

As in any study, there are several limitations that should be considered. Obviously, throughout this “in-the-field” study, not all research conditions could be controlled for 100% of the time, such as weather conditions involving T_*a*_ and the degree of solar radiation, the training, and the dietary management of participating horses. These factors may have affected the individual T_*sk*_ and T_*c*_ time profiles. However, this applies to all “in-the-field” competition studies and, under ideal conditions, should not interfere with the reliability of a solid thermoregulatory monitoring proxy suitable for assuring the thermoregulatory wellbeing of competition horses in the field (74). Endeavors to assess the thermal environmental variables were limited to BOM and HOBO recordings of the T_*a*_ and the RH, with the HOBO device placed at one location. Other essential external variables, such as wind speed, were not included in the T_*a*_ measurements (17). The current study involved only one type and location of the wearable T_*sk*_ sensor based on IR technology. In the future, other thermo-physical measuring approaches will prove to be more robust. However, on all occasions, the involvement of a validated “gold standard” against which the performance of new individual monitoring devices is set should be an essential part of future studies (75).

## Conclusion

While the method of monitoring T_*sk*_ may be non-invasive and straightforward, our results have clearly shown that T_*sk*_ monitoring on its own does not reliably estimate the T_*c*_ evolvement during a field exercise in endurance horses since a correlation between T_*c*_ and T_*sk*_ could not be identified. Notably, a high T_*sk*_ at a single point during a field exercise in a cool T_*a*_ did not identify the endurance horses with an increased T_*c*_. Further research into T_*c*_ monitoring in different equine sports and under differing weather conditions must be undertaken to create a baseline for further fine-tuning hot weather policies. Accordingly, veterinarians, trainers, and owners can be advised to continuously monitor T_*c*_ to ensure the health and welfare of all horses.

## Data Availability Statement

The raw data supporting the conclusions of this article will be made available by the authors, without undue reservation.

## Ethics Statement

The animal study was reviewed and approved by University of Adelaide Animal Ethics Committee. Written informed consent was obtained from the owners for the participation of their animals in this study.

## Author Contributions

E-LV prepared and carried out the study design, data collection, preparation and creation of the database, descriptive and part of statistical analysis, interpretation of the data, and writing of the manuscript. CD was involved in study design, preparation and creation of the database, analysis and interpretation of the data, and writing of the manuscript. GH contributed to drafting and revising the manuscript. TM contributed to reviewing the manuscript and statistical analysis. The final manuscript was read and approved by all authors.

## Funding

This study was partly financially supported by a grant from the University of Adelaide for the cost of monitoring equipment.

## Conflict of Interest

The authors declare that the research was conducted in the absence of any commercial or financial relationships that could be construed as a potential conflict of interest.

## Publisher's Note

All claims expressed in this article are solely those of the authors and do not necessarily represent those of their affiliated organizations, or those of the publisher, the editors and the reviewers. Any product that may be evaluated in this article, or claim that may be made by its manufacturer, is not guaranteed or endorsed by the publisher.

## Footnotes

^1^ https://connect.garmin.com/

^2^ www.graphpad.com

## Abbreviations

GI: Gastrointestinal
HR: heart rate
bpm: beats per minute
B.O.M.: Bureau of Meteorology
GPS: Global Positioning System
A.E.R.A.: Australian Endurance Riders Association
SAERA: South Australian Endurance Riders Association.
