# Supplementary material for: Is Continuous Monitoring of Skin Surface Temperature a Reliable Proxy to Assess the Thermoregulatory Response in Endurance Horses During Field Exercise?
Source: Front Vet Sci. 2022 May 27;9:894146. doi: 10.3389/fvets.2022.894146 (PMC9196037; doi:10.3389/fvets.2022.894146)
Supplement: Supplementary file 1 [file Data_Sheet_1.PDF]

frontiers in Physiology

ORIGINAL RESEARCH
published: 26 August 2021
doi: 10.3389/fphys.2021.708737

# Continuous Monitoring of the Thermoregulatory Response in Endurance Horses and Trotter Horses During Field Exercise: Baselining for Future Hot Weather Studies

*Elisabeth-Lidwien J. M. M. Verdegaal[1,2,3]*, Gordon S. Howarth[2], Todd J. McWhorter[2], Berit Boshuizen[3], Samantha H. Franklin[1,2], Carmen Vidal Moreno de Vega[3], Stacey E. Jonas[2], Louise E. Folwell[2] and Catherine J. G. Delesalle[3]*

*[1] Equine Health and Performance Centre, University of Adelaide, Adelaide, SA, Australia, [2] School of Animal and Veterinary Sciences, Roseworthy Campus, University of Adelaide, Adelaide, SA, Australia, [3] Research Group of Comparative Physiology, Department of Virology, Parasitology and Immunology, Faculty of Veterinary Medicine, Ghent University, Ghent, Belgium*

OPEN ACCESS

***Edited by:***
*James (Jim) David Cotter,*
*University of Otago, New Zealand*

***Reviewed by:***
*Jane Williams,*
*Hartpury University and Hartpury College, United Kingdom*
*Duncan Mitchell,*
*University of the Witwatersrand, South Africa*

****Correspondence:***
*Elisabeth-Lidwien J. M. M. Verdegaal*
*lidwien.verdegaal@adelaide.edu.au*

***Specialty section:***
*This article was submitted to Exercise Physiology, a section of the journal Frontiers in Physiology*

***Received:*** *12 May 2021*
***Accepted:*** *29 July 2021*
***Published:*** *26 August 2021*

***Citation:***
*Verdegaal E-LJMM, Howarth GS, McWhorter TJ, Boshuizen B, Franklin SH, Vidal Moreno de Vega C, Jonas SE, Folwell LE and Delesalle CJG (2021) Continuous Monitoring of the Thermoregulatory Response in Endurance Horses and Trotter Horses During Field Exercise: Baselining for Future Hot Weather Studies. Front. Physiol. 12:708737. doi: 10.3389/fphys.2021.708737*

Establishing proper policies regarding the recognition and prevention of equine heat stress becomes increasingly important, especially in the face of global warming. To assist this, a detailed view of the variability of equine thermoregulation during field exercise and recovery is essential. 13 endurance horses and 12 trotter horses were equipped with continuous monitoring devices [gastrointestinal (GI) pill, heartrate (HR) monitor, and global positioning system] and monitored under cool weather conditions during four endurance rides over a total of 80 km (40 km loops) and intense trotter track-based exercise over 1,540 m. Recordings included GI temperature ($T_C$), speed, HR and pre- and post-exercise blood values. A temperature time profile curve of $T_C$ was constructed, and a net area under the curve was calculated using the trapezoidal method. Metabolic heat production and oxygen cost of transport were also calculated in endurance horses. Maximum $T_C$ was compared using an independent samples *t*-test. Endurance horses (mean speed 14.1 ± 1.7 km $h^{-1}$) reached mean maximum $T_C$ (39.0 ± 0.4°C; 2 × 40 km in 8 horses) during exercise at 75% of completion of $T_C$ exercise and $T_C$ returned to baseline within 60 min into recovery. However, the mean $T_C$ was still 38.8 ± 0.4°C at a HR of 60 bpm which currently governs "fit to continue" competition decisions. Trotters (40.0 ± 2.9 km $h^{-1}$) reached a comparable mean max $T_C$ (38.8 ± 0.5°C; 12 horses) always during recovery. In 30% of trotters, $T_C$ was still >39°C at the end of recovery (40 ± 32 min). The study shows that horses are individuals and thermoregulation monitoring should reflect this, no matter what type of exercise is performed. Caution is advised when using HR cut-off values to monitor thermal welfare in horses since we have demonstrated how $T_C$ can peak quite some time after finishing exercise. These findings have implications for training and management of performance horses to safeguard equine welfare and to maximize performance.

**Keywords: thermoregulation, hyperthermia, gastrointestinal pill, endurance, trotters, metabolic heat (H), recovery, exercise**

## INTRODUCTION

Exertional heat illness (EHI) in horses is characterized by severe central nervous system dysfunction, such as physical collapse, and has been described in detail (Brownlow et al., 2016; Brownlow and Mizzi, 2020). Recently, a Japanese study identified a wet-bulb globe temperature (WBGT) index above 28°C to be responsible for a 28.5% higher risk for development of EHI when compared to the index below 20°C (Takahashi and Takahashi, 2020). A progressive increase in the prevalence of human EHI casualties is anticipated due to global warming (Raymond et al., 2020) and a similar effect can be anticipated for animals in general. In continents with warm climates and established horseracing industries, a WBGT above 28°C is often recorded during approximately 2–3 months annually. These regions include the southern part of North America and Europe, Oceania, parts of Africa, South America, and Asia (Raymond et al., 2020). It is a common misconception that EHI affects horses in only very hot and humid weather conditions, however, EHI casualties are also reported to occur on warmer pre-season days. To date, EHI has been underreported most probably because only the more severe and overt clinical cases are being recognized, leaving many mild cases unnoticed with possibly deleterious effects (Brownlow et al., 2016; Brownlow and Mizzi, 2021).

Consequently, there is great need to further fine-tune early detection of hyperthermia and to obtain a detailed view of the flexibility of equine thermoregulation in the field under different exercise and climatic conditions. Such knowledge will allow for identification of predisposing factors that may lead to the formulation of evidence-based hot weather policies and recommendations for the post-exercise and recovery period to prevent hyperthermia, EHI and EHS (Hosokawa et al., 2019; MOOC heat illness, 2021). Another critical aspect that needs to be clarified is the optimal rest and recovery period that is required before "return to exercise" is allowed after an EHI event. For example, human athletes who have experienced an EHI episode are required to rest and rehabilitate for a certain period, varying from 3 to 6 weeks to a year (Adams and Jardine, 2020). Similarly, various regulations govern eliminated endurance horses who are given rest periods to recover following a competition (AERA, 2020; FEI, 2021).

The way the thermoregulatory system is challenged greatly depends on the speed and distance that are realized by the exercising horse (Hodgson et al., 1994). Additionally, important breed differences are to be expected (Fielding et al., 2011). For example, trotter horses are bred for harness racing over distances ranging from 1,540 m (short distance races), 2,140 m (medium distance races), 2,640 m (long distance races), and rarely to 3,140 m (ultra-long-distance races), with average race speeds of 47.3–48.6 km $h^{-1}$ (Bertuglia et al., 2014). In comparison, endurance horses compete over distances of 80–160 km divided over exercise loops (sections) of 30–40 km under the regulations of either the National Endurance Riding Associations or the Fédération Équestre Internationale (FEI, 2018; FEI, 2021). The range of mean winning speeds at FEI 100–120 km endurance rides around the world varies between 14.1 and 24.8 km $h^{-1}$ while reported maximum winning speeds range from 17.2 to 26.2 km $h^{-1}$ (Nagy et al., 2010). After each exercise loop, a recovery rest period is imposed to allow the HR to return to below values of 60 bpm under the regulations of the Australian Endurance Riding Association or 64 bpm as per FEI regulations (Nagy et al., 2012; FEI, 2018; AERA, 2020). Once this is achieved, a follow-up inspection including metabolic and gait assessment is performed by the certified endurance veterinarian to determine whether the horse is deemed fit to continue with the next exercise loop or qualifies for completion of the competition. Clinical assessment during endurance rides is essential to ensure sufficient recovery in all horses since a high elimination rate (nearly 50%) is reported, and more specifically, the elimination rate due to metabolic disorders in endurance exercise varies between 4.2 and 15% (Barnes et al., 2010; Nagy et al., 2010, 2014; Fielding et al., 2011, 2017; Younes et al., 2015; Bennet and Parkin, 2018; Legg et al., 2019). Common debilitating metabolic disorders include abdominal discomfort, dehydration, and exertional rhabdomyolysis (Fielding et al., 2009; Verdegaal et al., 2018; Misheff, 2020).

At present, little is known about the core body temperature evolvement in real time during different types of exercise performed by racehorses and endurance horses under field conditions. The vast majority of thermoregulatory studies have been conducted under indoor laboratory conditions using a treadmill and subjecting the horses to performance of specific standardized exercise tests. To this end, monitoring of arterial pulmonary blood temperature has proven to be "the gold standard" (Hodgson et al., 1993; Marlin et al., 1996; Courouce-Malblanc, 2013; Hodgson, 2014). However, this approach is generally not feasible and too invasive under field conditions; only one study reported similar invasive methods in three horses during free field exercise using thermistors to measure blood and brain temperature (Mitchell et al., 2006). A few studies are available that focus on continuous monitoring of equine thermoregulation during exercise and recovery in the field (Smith et al., 2006; Verdegaal et al., 2017). Smith et al. (2006) used intra-uterine temperature loggers as a non-surgical, minimally invasive method to measure evolvement of intra-uterine temperature during field exercise. This method has the advantage of collecting data over several competitions, however, it does not monitor real-time core body temperature as data were downloaded after removal of the logger. Obviously, the intra-uterine method is only applicable in mares. No data are available on the long-term effect of these loggers on uterine function. In contrast, our telemetric recording method is able to provide real-time results and does not require subsequent time-critical analysis. This could be undertaken at any time after completion of the exercise period (Verdegaal et al., 2017).

It is important to note that most existing field studies compare rectal temperature ($T_{re}$) before and after exercise (Kohn and Hinchcliff, 1995; Kohn et al., 1995; Hargreaves et al., 1999; Jeffcott et al., 2009; Wallsten et al., 2012). However, $T_{re}$ is reported to significantly lag behind the core body temperature both during

**Abbreviations:** GI, Gastrointestinal; COT, Cost of transfer.

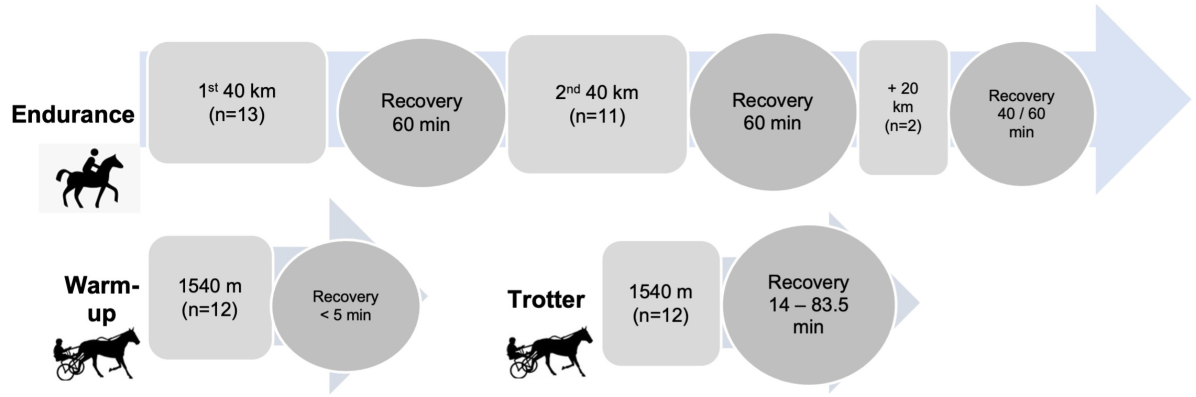


**FIGURE 1 |** Flow chart of study design describing two types of exercise and distance; km, kilometers; m, meters; n, number of horses; min, minutes; endurance exercise; moderate trotter warm-up exercise; and fast trotter exercise.

and after exercise (Hodgson et al., 1993; Geor et al., 1995; Verdegaal et al., 2017). This means that the heating of the core of the body is always reflected in the $T_{re}$ at a much later stage and at a lower temperature. Recently, we reported and validated a novel real-time temperature monitoring method namely a gastrointestinal (GI) pill that allows for accurate continuous recording of the GI temperature in exercising horses in the field (Verdegaal et al., 2017). The GI pill proved to be a more accurate and precise tool to monitor thermal response than serial $T_{re}$ measurements. Importantly, the GI temperature ($T_c$) was consistently and significantly higher than the $T_{re}$ (mean difference 0.3°C, with a range of 0.2–0.3°C) and $T_c$ increased earlier than $T_{re}$ on all occasions (Verdegaal et al., 2017).

The aim of the current study was to continuously monitor and compare the time profile of the dynamic thermoregulatory response of endurance horses in real-time competitions and trotter horses in the field. The GI temperature profiles were established during exercise and recovery under cool weather conditions to create a baseline thermal response profile for future field studies performed under hot and humid weather conditions. For this purpose, trotter and endurance horses were equipped with several non-invasive monitoring devices [GI pill ($T_c$), heartrate (HR) monitor, and global positioning system (GPS) monitor] to continuously monitor their physiologic responses to field exercise.

## MATERIALS AND METHODS

### Horses

In the first study group, 13 trained endurance horses (Horses 1–13) were enrolled, competing at either 40, 80, or 100 km distances. The study design is presented in a flowchart in **Figure 1**. The characteristics of the horses such as breed, age, sex, body mass, coat color, together with competition distance are presented in **Table 1**. The body mass of endurance horses was determined using an equine mass tape (Horse and pony weight tape®; Wagner and Tyler, 2011). When calculating the energy expenditure of the horses, the estimated mass of the rider was considered (Pagan and Hintz, 1986) based on "rider riding division criteria" determined in the AERA rules (AERA, 2020). These criteria encompass: (1) HM division—heavy mass rider of 91 kg or greater, estimated average 103 kg; (2) MM division—middle mass of 73 kg or greater, estimated average 83 kg; (3) LM division—low mass, no minimum mass, estimated average 63 kg. The trotter horses were weighed on a mass scale (Ruddweigh 700®, Gallagher Group Limited, New Zealand).

All 13 endurance horses (Horses 1–13, **Table 1**) were sourced on a voluntary basis through the South Australian Endurance Riders Association (SAERA) at a maximum of four horses per event. All horse owners signed a written consent form before enrolment. The horses remained under the care of their owners for the duration of the events and owners could withdraw their horse at any time during the study. Before competition, all horses were subjected to a health inspection conducted by endurance veterinarians according to AERA riding rules (AERA, 2020).

The second study group consisting of 12 unconditioned trotter horses (Standardbreds; Horses 14–26, **Table 1**) was randomly selected from a university-based teaching herd at a maximum of four horses per event. Trotters were kept in large paddocks without additional exercise or training. Before enrolment, trotters were subjected to a standard clinical health check. The present study was approved by the University of Adelaide Animal Ethics Committee (project number S-2011-224), which conformed to the Australian Code of Practice for the care and use of animals for scientific purposes, Canberra, Australia, 2004.

### Study Design

The study design is illustrated in **Figure 1**. Endurance horses (**Table 1**) were competing at distances of either 40 km ($n$ = 2) or 80 km ($n$ = 11) and two of those horses continued to cover 100 km. Data collection for endurance horses was performed during four endurance events during the cooler South Australian winter months (May, June, and July) and event locations varied from −34°28′5.70′′ S to −34°32′3.08′′ S. The horses exercised

**TABLE 1 |** Study population characteristics and its monitoring devices.

| Horse number | Sex | Age (y) | Breed | Body mass (kg) | Total mass incl rider | Coat color | Distance (km) | Horse experience (years) | Age start (years) | GI Pill Y/N | GPS/HR Y/N | BOM (°C; $T_a$), min – max | PCV (Hct), lactate, TS, pH Y/N | Blood gas Y/N | Biochemistry (electrolytes, glucose) Y/N | Hematology |
|---|---|---|---|---|---|---|---|---|---|---|---|---|---|---|---|---|
| 1 | G | 11 | Arab | 669 | 752 | Gr | 80 | 6 | 4 | Y | Y | 13–26 | Y | Y | Y | N |
| 2 | G | 9 | Arab | 484 | 587 | B | 80 | 2 | 7 | Y | Y | 13–26 | Y | Y | Y | N |
| 3 | M | 13 | Arab | 426 | 489 | C | 80 | 3 | 10 | Y | Y | 6–19 | Y | Y | Y | N |
| 4 | M | 7 | QH × TB | 470 | 573 | C | 100 | 1 | 6 | Y | Y | 6–19 | Y | Y | Y | N |
| 5 | M | 11 | Arab | 450 | 533 | C | 80 | 2 | 9 | Y | Y | 6–19 | Y | Y | Y | N |
| 6 | M | 8 | Arab | 370 | 453 | Gr | 100 | 4 | 4 | Y | Y | 6–19 | Y | Y | Y | N |
| 7 | M | 9 | Arab | 450 | 523 | C | 80 | 2 | 7 | Y | N^ | 3–22 | Y | N | Y** | Y |
| 8 | G | 11 | Arab | 470 | 553 | Gr | 80 | 1 | 9 | Y | Y | 3–22 | Y | N | Y** | Y |
| 9 | G | 7 | QH | 490 | 573 | C | 80 | 1 | 6 | N | Y | 7–13 | Y | Y | Y | N |
| 10 | G | 10 | Arab × TB | 484 | 587 | C | 80 | 5 | 5 | N | Y | 7–13 | Y | Y | Y | N |
| 11 | G | 5 | Arab | 458 | 541 | B | 40 | 0 | 5 | N | Y | 7–13 | Y | Y | Y | N |
| 12 | G | 7 | Arab | 525 | 608 | B | 80 | NK | NK | N | Y | 3–22 | Y | N | Y** | Y |
| 13 | M | 15 | Arab | 480 | 563 | Gr | 40 | NK | NK | N | N^^ | 3–22 | Y | N | Y** | Y |
| 14 | G | 10 | SB | 484 | – | B | 1.54 | – | – | Y | Y | 5–13 | Y | N | N | N |
| 15 | G | 5 | SB | 610 | – | B | 1.54 | – | – | Y | Y | 5–13 | Y | N | N | N |
| 16 | G | 17 | SB | 482 | – | B | 1.54 | – | – | Y | Y | 5–13 | Y | N | N | N |
| 17 | G | 5 | SB | 604 | – | B | 1.54 | – | – | Y | Y | 5–13 | Y | N | N | N |
| 18 | G | 7 | SB | 554 | – | B | 1.54 | – | – | Y | Y | 3–18 | Y | N | N | N |
| 19 | G | 11 | SB | 482 | – | B | 1.54 | – | – | Y | Y | 6–14 | Y | N | N | N |
| 20 | G | 18 | SB | 480 | – | B | 1.54 | – | – | Y | Y | 6–14 | Y | N | N | N |
| 21 | G | 6 | SB | 602 | – | B | 1.54 | – | – | Y | Y | 6–14 | Y | N | N | N |
| 22 | M | 4 | SB | 508 | – | B | 1.54 | – | – | Y | Y | 14–20 | Y | N | N | N |
| 23 | G | 11 | SB | 482 | – | B | 1.54 | – | – | Y | Y | 14–20 | Y | N | N | N |
| 24 | G | 18 | SB | 480 | – | B | 1.54 | – | – | Y | Y | 14–20 | Y | N | N | N |
| 25 | G | 6 | SB | 602 | – | B | 1.54 | – | – | Y | Y | 14–20 | Y | N | N | N |

***Horse 1–13:** 13 endurance horses: G (n = 7), M (n = 6). Arab include part Arabian horses, QH, quarterhorse; TB, thoroughbred, endurance exercise; **Horse 14–25**: 12 untrained trotters, all Standardbred (SB): G (n = 12), fast trot exercise; G, gelding; M, mare. Gr, gray; C, chestnut; B, bay; The riders' and horses' performance history includes: age start, indicates age (years) when horse started competing; horse experience, indicates number of years in competition (40 km or more); GI pill, gastrointestinal pill; GPS, global positioning system; HR, heartrate monitor (Polar); BOM (2020); Bureau of Meteorology, local stations closest to location of exercise at varying km distance from the actual event in total 4 endurance locations and 1 trotter racetrack, distance ranged from 5.3 to 53 km; BOM stations were: Roseworthy 27.9 km distance from ride at Sandy Creek; Rosedale station 5.3 km from ride at Tarlee; Meningie station 53 km from ride at Coorong; Nuriootpa station 28.8 km from ride at Sedan; Roseworthy station at 2.2 km from trotter racetrack; N, no; Y, yes; NK, not known; ^indicates HR only; ^^indicates second 40 km only; blood gas analysis included pH, $pCO_2$, $pO_2$, $HCO_3^-$, base excess and lactate levels; **indicates biochemistry by VETSCAN (utilizes dry and liquid reagents); TS, total solids; TP, total protein; AST, Aspartate amino transferase; CK, creatinine kinase.*

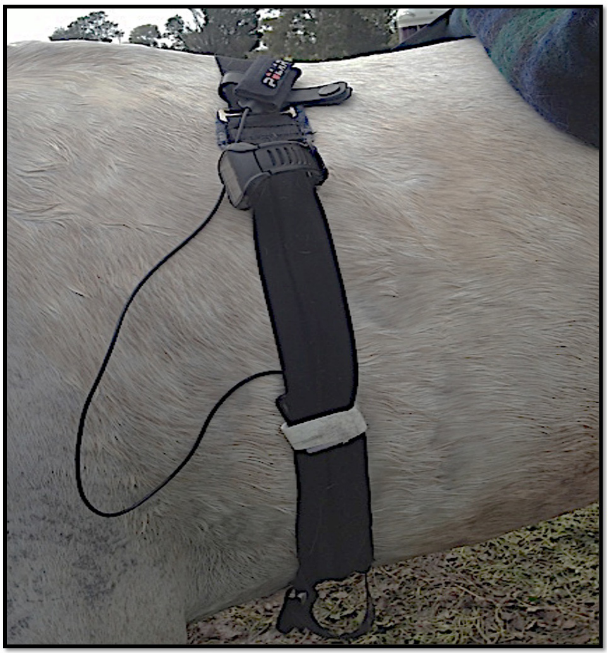

**FIGURE 2** | Monitoring equipment: A modified Equivital belt for use on horses with the external receiver device (Sensor Electronics Module) located inside of the belt at of the ventral part of the thorax to monitor the Tc by GI pill, while the Garmin GPS watch and the Polar heartrate electrodes were attached at the upper part of the belt.

under solar radiation through farming land of varying terrain with altitudes ranging from 4 to 462 m.

Following each exercise loop of 40 km, endurance horses were required to rest for 60 min according to AERA riding rules (AERA, 2020) and were subsequently inspected by independent endurance veterinarians before competition could be continued. Following every exercise loop, blood samples were collected. Subsequently, horses were cooled by pouring buckets of water over their bodies and then scraping it off. Horses were allowed to drink water and eat hay *ad libitum*.

The trotters were harnessed to a jogger and exercised under full sunlight over a 770 m sand training track in South Australia (−34°32′3.08″ S) during Australian winter months (June, July). The racetrack was harrowed and watered on a regular basis to keep the track conditions as constant as possible. Trotters were subjected to three consecutive phases of exercise: slow warm-up phase (770 m at 25 km $h^{-1}$, not included in data analysis); moderate warm-up phase: 1,540 m at a speed of 35 km $h^{-1}$ (9.7 m $s^{-1}$); and the more intense fast trotter exercise: 1,540 m at a speed of 42–50 km $h^{-1}$ (11.7–13.9 m $s^{-1}$; **Figure 1**). During the last phase, trotters were encouraged to exercise at their individual maximum speed. Post-exercise, blood samples were collected followed by cooling with a water hose for approximately 10 min and horses were allowed to drink water *ad libitum*. The $T_c$ recovery period varied from 32 to 83 min depending on the duration of the individual $T_c$ recovery.

## Continuous Monitoring of GI Temperature Over Time

All horses were equipped with monitoring equipment (**Table 1**). The GI $T_c$ representing the core temperature was recorded continuously during exercise and recovery using the ingestible telemetric GI pill (Jonah pill®; Phillips, United States. The night before undertaking exercise, GI pills were successfully administered to eight endurance horses and 12 trotters using nasogastric intubation as previously described (Verdegaal et al., 2017). The timing of the GI pill administration was random, unrelated to the horses' feeding regime.

Temperature was recorded by an external receiver, Equivital® Sensor Electronics Module (SEM, EQ02 Equivital data Logger®, Hidalgo, United Kingdom. The receiver was positioned in a pocket of the Equivital Sensor Belt®. This system was modified to accommodate a sturdy strap fitted closely around each horse's saddle girth (**Figure 2**). Equivital $T_c$ data were recorded every 15 seconds and were uploaded from the SEM to the Equivital Software Manager® for data processing.

## Monitoring of Distance, Speed, Inclination, and Heartrate Over Time

Traveled distance, speed, and inclination of the course were recorded every second using a GPS monitor (Garmin Forerunner 910XT GPS watch®; Garmin Ltd., Schaffhausen, Switzerland) attached to the gullet of the saddle. The HR was continuously monitored telemetrically by the Garmin watch using Polar electrodes (Polar Electro®, Kempele, Finland) and recorded every second (Parker et al., 2009). The GPS and HR data were uploaded from the Garmin watch to the Garmin Connect and Garmin Training Centre[1].

## Ambient Environment

On each data collection day, ambient temperature ($T_a$) was recorded continuously in a shaded area of the rest area (post-exercise) using a data logger device (Onset HOBO Pro V2 logger temp U23-00®, Onset Computer Corporation, Bourne, ME, United States). In addition, $T_a$ data were obtained from the Bureau of Meteorology as presented in **Table 1**.

## Blood Sampling and Analysis

Blood samples from endurance horses were analyzed using portable equipment in the field and data are presented in **Table 1**. Venous blood was collected (by SJ, E-LV, or LF) from the left jugular vein by needle and divided in two separate syringes within 3 min after exercise. The first blood sample was collected in a 3 ml syringe containing heparin and closed with an airtight seal for venous blood gas analysis using a portable blood gas analyzer (EPOC, Epocal®; Ottawa, Canada; Averay et al., 2014; Kirsch et al., 2019). The second venous blood sample was collected in a 20 ml syringe and distributed into a lithium heparin blood tube and a plain blood tube and stored on ice before samples were centrifuged at 3,000 *g* for 10 min (Hematocrit centrifuge 200®; Hettich Lab, Tuttlinger, Germany). In trotters, venous blood

[1] https://connect.garmin.com/

**TABLE 2 |** Overall variables during exercise and recovery of endurance and trotter exercise.

| Type exercise | Endurance ($n$ = 16 40 km) | Trotter 2nd warm-up ($n$ = 12 1,540 m) | Trotter ($n$ = 12 1,540 m) |
|---|---|---|---|
| Duration (min) exercise | 198 ± 63 (83–247; 40 km, $n$ = 25); $T_c$ only: 191 ± 43 ($n$ = 16) | 3.7 ± 0.4 | 2.9 ± 1.4. (1.5–6.1) |
| Duration (min) recovery | 60 | 4.9 ± 1.4 (2.6–7) | 40.2 ± 30.2 (11.5–83) |
| $T_c$ (°C) | 38.5 ± 0.3 (38.2–39.0) | 37.9 ± 0.3 (37.5–38.3) | 38.1 ± 0.3 (37.5–38.5) |
| Max $T_c$ (°C; during exercise or recovery) | 39.0 ± 0.4 (38.5–39.9**) | 38.0 ± 0.3** | 38.8 ± 0.5 (37.6–39.3)** |
| Time to max $T_c$ (min) | 143 ± 60 (54 – 245, $n$ = 15) | NA** | 34.3 ± 28.4 (6.5–79)** |
| Exercise distance (km) to max $T_c$ | 29.5 ± 11.6 (11–40, $n$ = 15) | NA | NA |
| Max $T_c$ ≥ 39°C | 8/16 | 0 | 4/12 |
| Max $T_c$ ≥ 38.5°C | All | 0 | 5/12 |
| Speed at max $T_c$ (km $h^{-1}$) | 16.1 ± 4.3 (4.7–21) | 22.5 ± 8.3 | 337.7 ± 10.0 (18.6–51.9) |
| HR at max $T_c$ (bpm) | 105 ± 29 (63–152) | 135.1 ± 39.0 | 145 ± 36 (80–204) |
| Return $T_c$ to 38.5°C | 15/16 | 6/12 | 1/12 (and 1 $T_c$ never above 38) |
| Time max $T_c$ to 38.5°C (min) | 21.2 ± 22.9 (0–80) | NA | NA |
| Return $T_c$ to 38.3°C | 13/16 | 2/12 | 1/12 (and 1 $T_c$ never above 38) |
| Time max $T_c$ to 38.3°C (min) | 31 ± 24.6 (0 ->77; $n$ = 13/16) | NA | NA |
| Return $T_c$ to 38.0°C | 10/16 | 8/12 | 8/12 (1 $T_c$ never above 38) |
| Time max $T_c$ to 38.0°C (min) | 39.6 ± 14 ($n$ = 8/16) | 0 (6//12; 2 $T_c$ never above 38) | NA |
| Return to base $T_c$ (Y/N) | 4/16 | 8/12 | 1//12 (and 1 $T_c$ never above 38) |
| Time max $T_c$ to baseline (min) | 52 ± 37 ($n$ = 4/16) | 0 (4/12); (1 $T_c$ never above baseline) | NA |
| Net AUC $T_c$ exercise (endurance: 2nd 40 km from base $T_c$) | 7,116 ± 1,997 (7,203 ± 3,125) | 7.9 ± 11 | 4.8 ± 8.5 |
| Net AUC $T_c$ exercise 1st 40 km | 77,421 ± 1,940 | NA | NA |
| Net AUC $T_c$ exercise 2nd 40 km | 6,811 ± 2,138 | NA | NA |
| Net AUC $T_c$ recovery | −1,516 ± 1,326 ($n$ = 15) | 5.4 ± 6.8 | 1,821.3 ± 4,434.0 (−14.3–15,769) |
| Net AUC $T_c$ recovery 1st 40 km | −1,979 ± 1,555 ($n$ = 7) | NA | NA |
| Net AUC $T_c$ recovery 2nd 40 km | −1,169 ± 1,102 ($n$ = 8) | NA | NA |
| Delta $T_c$ first 10 min recovery (°C) | −0.24 ± 0.34 (−0.24–0.15, $n$ = 15) | NA | + 0.36 ± 0.16 (0–0.58; $n$ = 11) |
| Net AUC $min^{-1}$ (°C $min^{-1}$) exercise | 37.3 ± 9.9 | 2 ± 2.7 | 1.9 ± 3.7 |
| Delta $T_c$ end exercise to end recovery | −0.71 ± 0.4 (−1.16–0.23) | 0.1 ± 0.1 (0–0.2) | 0.6 ± 0.4 (0–1.3) |
| Delta $T_c$ end exercise to end recovery $min^{-1}$ | −0.01 ± 0.01 (−0.02–0.02) | 0.02 | 0.04 ± 0.02 (0–0.06) |
| Time to HR ≤ 60 bpm (min) | 6.3 ± 6.5 (0–24) | 2.3 ± 1.2 (0.5–4) | 23.1 ± 20.9 (5–51) 4/12 |
| $T_c$ > 39°C at end recovery | 1/16 (39.13 after 90 min) | NA | 4/12 |
| Net AUC $min^{-1}$ (°C $min^{-1}$) recovery | −23.7 ± 20.4 (−55–13.8) | 2.9 ± 3.3 | 54.5 ± 143.6 |
| Speed (km $h^{-1}$) | 14.1 ± 1.7 (10.5 −17.4; $n$ = 22, without horse 7) | 34.1 ± 3.5 (30.4–38.0; $n$ = 7) | 38.2 ± 0.3 (38.0–38.8; $n$ = 7) |
| Max speed (km $h^{-1}$) | 23.4 ± 2.8 (19.2–30; $n$ = 22) | 34.1 ± 7.3 (25.0–42.3; $n$ = 7) | 49.7 ± 3.9 (42–52.7; $n$ = 7) |
| HR (bpm) | 114 ± 13 (89 −143; $n$ = 24) | 130 ± 32 (75–177; $n$ = 8) | 151 ± 17 (130–169; $n$ = 8) |
| H (kJ) total 80 km exercise# | 71,000 ± 12,000 (52,000–94,000; $n$ = 22) | – | – |
| COT (ml $O_2$ $kg^{-1}$ $m^{-1}$)# | 0.13 ± 0.01 (0.11–0.15; $n$ = 22) | – | – |
| $\dot{V}O_2$ ($LO_2$ $min^{-1}$) # | 15.0 ± 2.3 (11.2–20.5; $n$ = 22) | – | – |

*Data are presented as overall mean ± SD. $T_c$, GI pill temperature; n, number identified only if different to 16 or 12 exercise periods; max $T_c$, maximum $T_c$; AUC, area under the curve; #indicates calculated; HR, heartrate; H, metabolic heat production; COT, cost of transfer; $\dot{V}O_2$ L $min^{-1}$, oxygen consumption in liters per min; *indicates during exercise; **indicates during recovery; NA, not applicable; –indicates not available.*

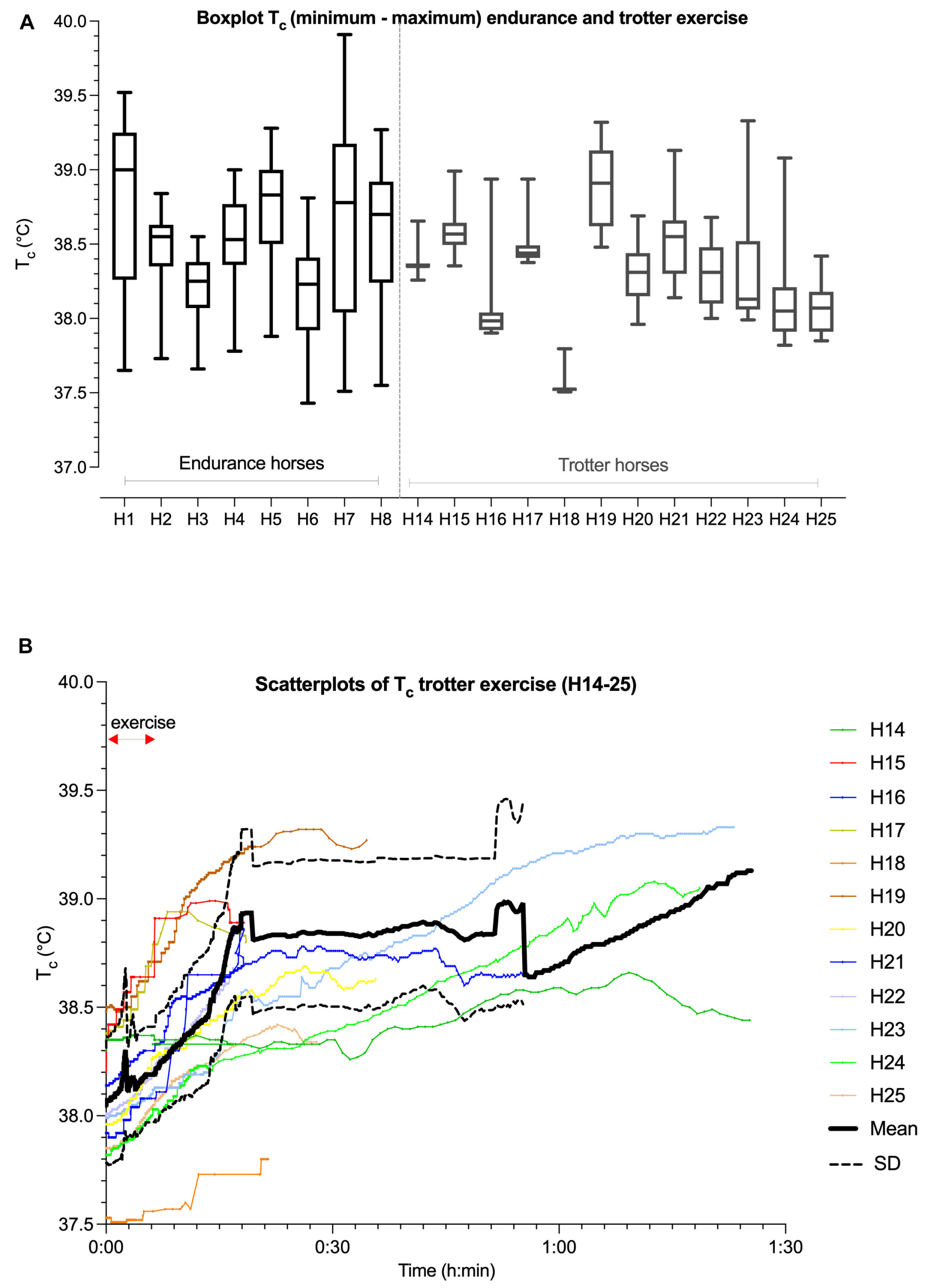


**FIGURE 3 |** Box and whisker plots demonstrating median (solid line), interquartile range (identified by rectangular area), and minimum to maximum (max) ranges of data (whiskers or error bars) of boxplots of $T_c$ (°C) in horses (H; horse number) in endurance (H1–H8) and trotter (H14–H25) exercise **(A)**. Scatterplots with $T_c$ (°C; left *y*-axis) over time [duration of exercise in hours (h) and minutes (min)] (*x*-axis) in trotter horses (H14–25) and its mean (thick black line) ± SD (dotted black line, calculated when $n > 3$ horses) during high intensity trotter exercise over time **(B)**.

samples were collected from the left jugular vein by a vacutainer technique in lithium heparin tubes (presented in **Table 1**).

Hematocrit was determined with the microhematocrit method and plasma total solids via a refractometry meter [Refractometer, RHC-200 (ATC)®]. Plasma lactate concentration was analyzed with a hand-held lactate analyzer (Accutrend Plus®, Roche, United States). Blood pH recording in trotters was measured using approximately 2 ml of blood and a pH meter (pH Cube®; TPS Pty Ltd, Brendale, Australia) with a pH calibration prior to every measurement by a voltage plot against the specified pH value of the buffer solution followed by introduction of the probe into the blood for approximately 30 s to read the pH. The biochemistry profiles were analyzed using a blood gas analyzer EPOC® [$n$ = 9; sodium ($Na^+$), potassium

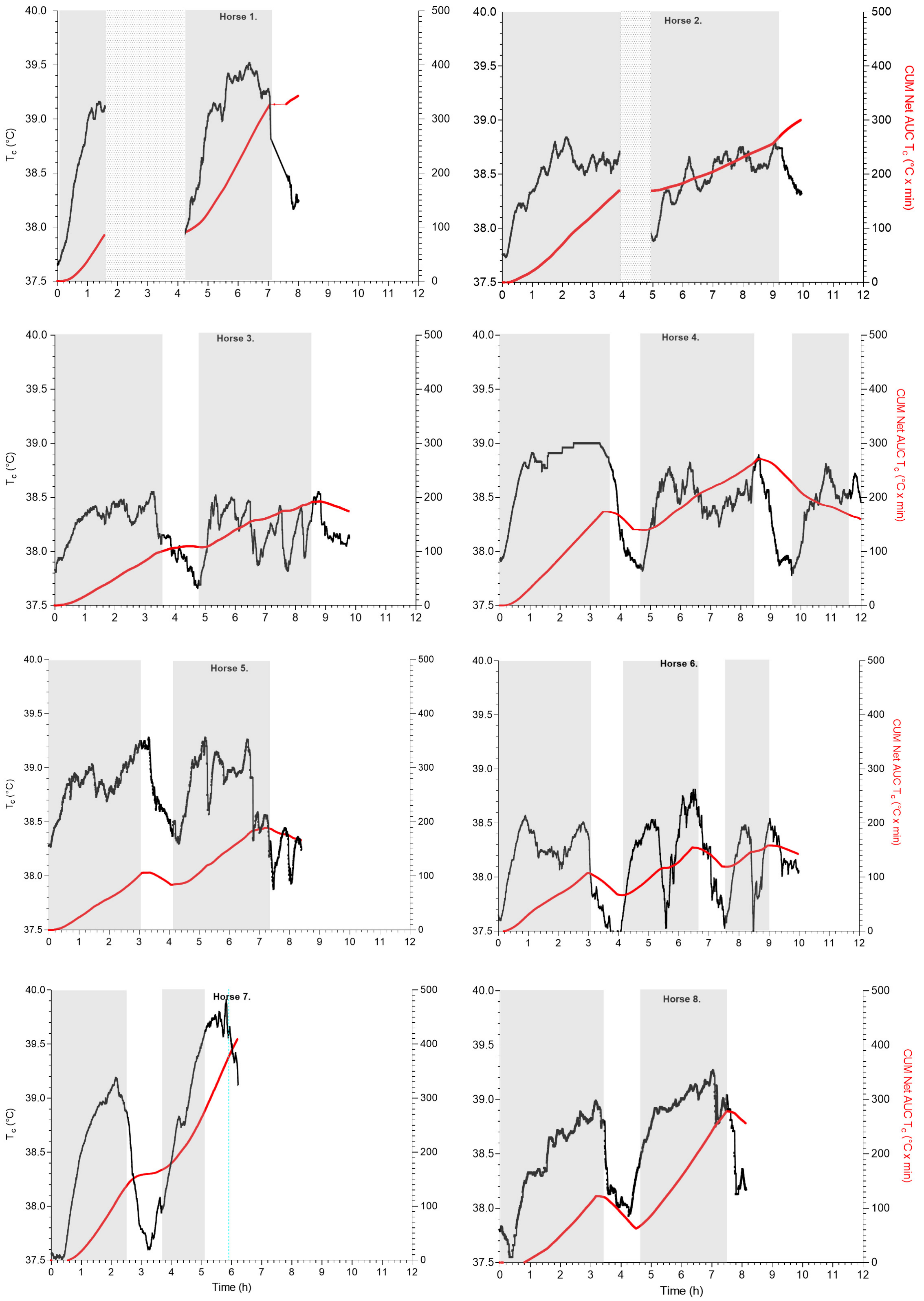


**FIGURE 4 |** Scatterplots of $T_c$ (°C; left *y*-axis, black line) and cumulated net AUC ($T_c$°C × min; right *y*-axis, red line) over time (hours, h) during $T_c$ exercise periods (identified as gray blocks) and recovery in endurance horses H1–8; H1 data loss at end of leg 1 and H1, H2 without $T_c$ recording during the first recovery (identified as spotted blocks); H4, H6 continued to 100 km total.

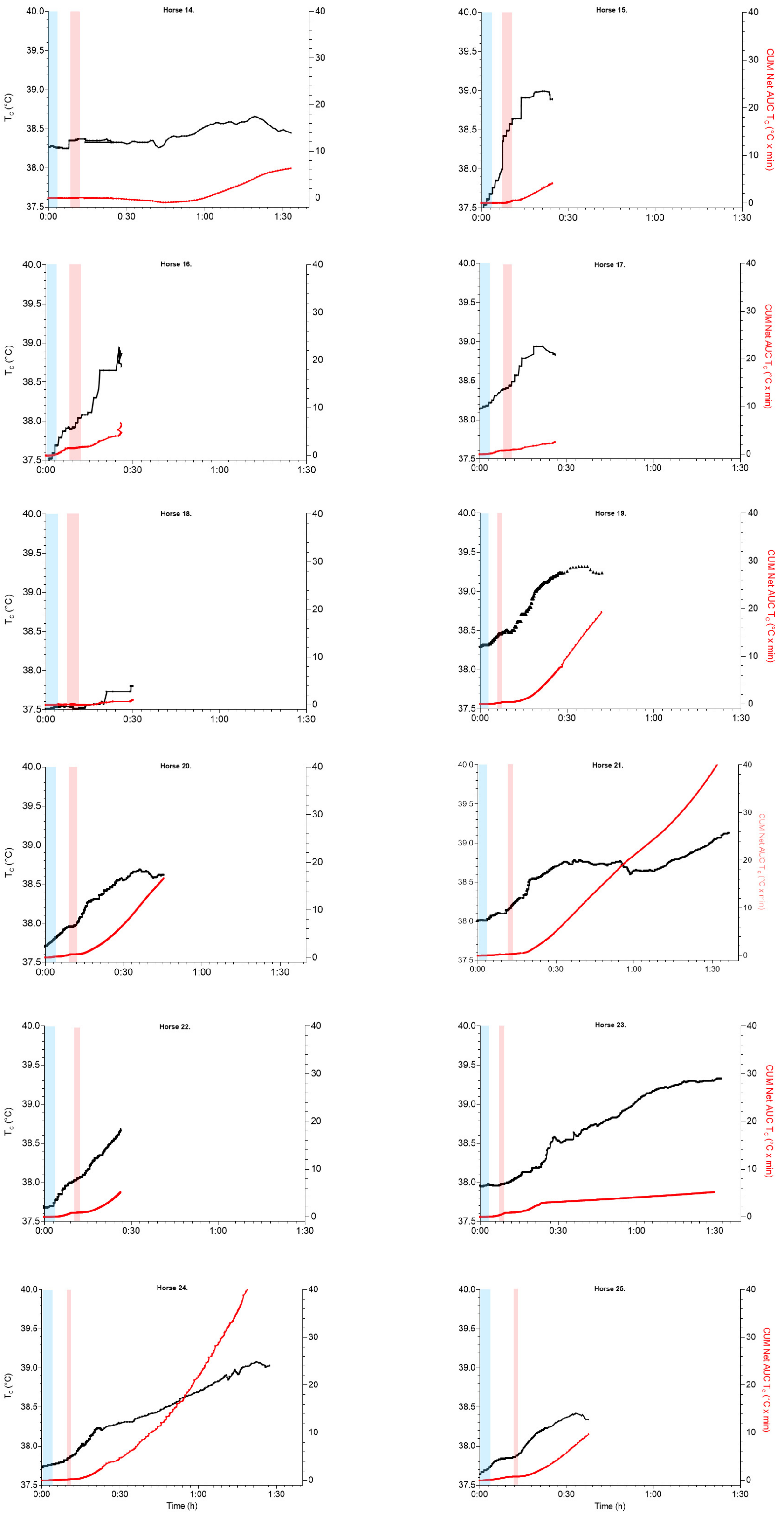


**FIGURE 5 |** Scatterplots of $T_C$ (°C; left *y*-axis, black line) and cumulated net AUC ($T_C$, °C × min; right *y*-axis, red line) over time (hours, h) during exercise and recovery in trotter horses H14–25, warm-up exercise (moderate intensity, identified as blue blocks), and trotter exercise (high intensity, identified as light-red blocks).

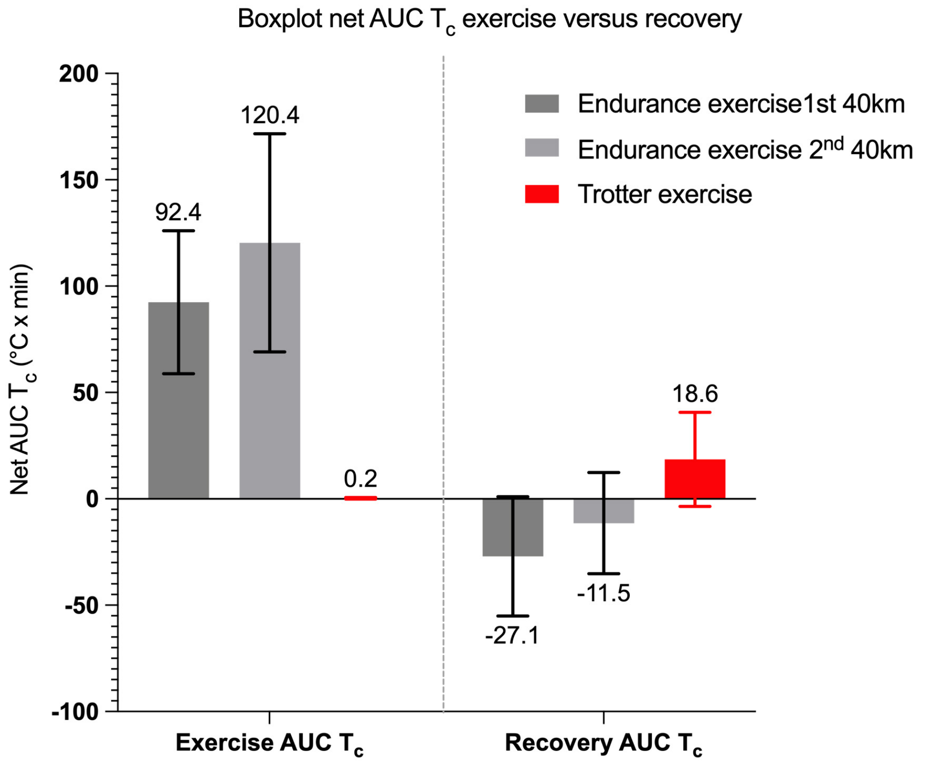


**FIGURE 6 |** Boxplot of the dynamic thermal response presented by net AUC $T_c$ (°C × min) during $T_c$ exercise period of endurance and trotter exercises (presented with mean net AUC on top bar). The 1st 40 km of endurance exercise was not significantly different to the 2nd 40 km. Net AUC $T_c$ of exercise and recovery from full speed trotter exercise over 1,540 m was minor during exercise while $T_c$ increased post-exercise as represented by positive net AUC $T_c$.

($K^+$), calcium ($Ca^{2+}$), glucose] or a dry biochemistry analyzer (Vetscan VS2®; Chemistry Analyzer, Abaxis, United States; $n = 4$; $Na^+$, $K^+$, $Ca^{2+}$, glucose, creatinine, urea, aspartate amino transferase, creatinine kinase). Hematology tests were performed in four horses (VETSCAN HM5 Hematology Analyzer®, Abaxis, United States). An overview of the blood analysis performed in individual horses is presented in **Table 1**.

## Data Processing

Each exercise period of 40 km (endurance) or 1,540 m (trotter) will be referred to throughout the study as the $T_c$ exercise period. Each recovery period (post-exercise) following 40 km (endurance) or 1,540 m (trotters) will be referred to as $T_c$ recovery period. Recordings performed during each $T_c$ period included GI temperature ($T_c$), speed, HR and post-exercise blood values. Hyperthermia was defined as $T_c$ above 39°C based upon the results of our previous study (Verdegaal et al., 2017) in which the mean maximum $T_c$ was 38.9°C (95% CI, 38.8–38.9°C) at 6 min post-exercise. A $T_c$ time profile curve of the individual horse was constructed from the endurance exercise, warm-up exercise (trotters) and (fast) trotter exercise. This included assessment of the value and time point of the maximum $T_c$ (max $T_c$) during the $T_c$ exercise or recovery period (40 km, total 80 km, or 1,540 m), the HR and speed at max $T_c$, recovery time (min) from max $T_c$ to a $T_c$ of 38.5, 38.3, and 38.0°C, respectively, and recovery to the baseline $T_c$ and number of horses with hyperthermia. In addition, the delta $T_c$ (°C change post-exercise) and the recovery time from max $T_c$ to a HR of 60 bpm were calculated as per Australian regulations (AERA, 2020).

To quantify the $T_c$ response, the net area under the $T_c$ curve (net AUC) was calculated for $T_c$ and summated for the total $T_c$ exercise period and the total $T_c$ recovery period (Horswill et al., 2008; Datta et al., 2021). The net AUC (baseline set at rest $T_c$) was calculated using the trapezoidal method of $T_c$ over time (min) expressed as °C × min. In this study, the net AUC is summated to present the cumulative $T_c$ – time distribution (Datta et al., 2021). The net AUC provides an estimate of the dynamic thermal response to thermal load. The thermal load takes into account exercise intensity, $T_a$ including solar radiation, and environmental evaporative power during exercise and recovery (IUPS Thermal Commission, 2001). Further processing of the recorded data included calculation of the metabolic heat production (H, kJ $min^{-1}$; IUPS Thermal Commission, 2001) and the oxygen cost of transport (COT—the oxygen consumption necessary to travel 1 m) for each endurance horse (Schroter and Marlin, 2002). Calculation of both H and COT provides a multi-perspective view on thermoregulatory challenges represented by different types of exercise. The following formula was used:

$$H = \dot{V}O_2 \times k \times \text{duration of exercise}$$

— in which $\dot{V}O_2$ represents the oxygen consumption rate expressed in liters per min, $k$ stands for heat liberated per liter of oxygen consumed (5 kcal; 21 kJ; Hodgson et al., 1993; McCutcheon and Geor, 2008) and duration of exercise is expressed in min. The rate at which oxygen is consumed in an exercising horse provides a direct indication of its metabolic rate and subsequent heat production (Hodgson et al., 1993). In the current study, $\dot{V}O_2$ was estimated by calculating the COT, speed and body mass as follows:

$$\dot{V}O_2 = \text{COT} \times \text{speed} \times \text{body mass}$$

— in which COT is the oxygen cost of transport in ml oxygen per kg body mass per m (ml $O_2$ $kg^{-1}$ $m^{-1}$), speed is expressed in m $min^{-1}$ and body mass in kg.

The COT is calculated based on an incline, decline, or flat terrain using the following equation (Schroter and Marlin, 2002):

$$\text{Uphill (incline) and flat terrain:}$$
$$\text{COT} = 0.123 + 1.561\,(\text{gradient})$$
$$\text{Downhill terrain (decline):}$$
$$\text{COT} = 0.123 + 1.591\,(\text{gradient}) + 9.762\,(\text{gradient})^2 + 14.0\,(\text{gradient})^3.$$

The gradient of the terrain was recorded as meters incline or decline indicated by the GPS device. The overall H per 40 km $T_c$ exercise period was calculated by cumulating the H and presented as cumulated H.

## Statistical Analysis

All data are presented as mean ± SD (range). Comparison and correlation analyses were performed using IBM SPSS Statistics 26.0 software or using GraphPad Prism version 9.1.2 for MacOS, GraphPad Software, San Diego, CA, United States[2]. The net AUC (°C × min) and max $T_c$ (°C) between the first and second 40 km exercise loops were compared using a paired $t$-test and a general linear model ANOVA approach which included horse ID as a random variable. The mean max $T_c$ was than compared between endurance and trotter exercise using an independent samples $t$-test. The correlation between time to HR ≤ 60 bpm (dependent) and the $T_c$ (°C) at end of exercise (independent) was assessed by a scatterplot and analyzed using a regression model. Blood values were compared using a one-way ANOVA *post hoc* Tukey's test. Statistical significance was set at $\alpha < 0.05$.

# RESULTS

All horses completed their exercise trial without any adverse events. No horses were withdrawn by owners. The owner of horse 13 erroneously removed the GPS equipment during the first 40 km. The GI pills were administered successfully to all horses resulting in complete $T_c$ recordings. Unfortunately, an exceptional event of a malfunctioning batch of GI pills prevented activation of the GI pill in 5 of the 13 endurance horses. The GPS data were not recorded (only the HR) in 1 endurance horse (horse 7) and in 1 trotter (horse 19) while HR was not recorded in horse 21 (**Table 1**). Unphysiological and unrealistic values (artifacts) for $T_c$, GPS and HR (below 10 bpm and above 220 bpm) were removed prior to analysis. The endurance horse group encompassed five mares and seven geldings of different breeds (although mainly Arabians) with an age of 9.5 ± 2.8 years and body mass of 479 ± 68 kg (**Table 1**). The mean age of starting endurance competitions was 6.1 ± 2.1 years with a mean of 2.5 ± 1.9 years of experience in endurance ride competitions. The $T_c$ was recorded in eight horses over a total of 80 km $T_c$ exercise separated in loops of 40 km and associated $T_c$ recovery periods of 60 min. The Equivital belt became dislodged in horse 1 during the first loop causing data loss at the end of a 40 km loop. As a result, the belt underwent additional modification for the subsequent recordings by fitting sturdy straps sandwiched into the belt to stabilize the girth position. The $T_c$ was not recorded during recovery following the first loop in horses 1 and 2 due to premature removal of the belt by the owners. The group of 12 trotter horses included 1 mare and 11 geldings with an age of 9.8 ± 5.3 years and body mass of 537 ± 60 kg (**Table 1**).

## Ambient Environment

On all occasions, the $T_a$ was relatively cool: mean minimum 6.7 ± 0.4°C, mean maximum 18.4 ± 2.9°C (**Table 1**). More specifically, the $T_a$ on the four separate days of endurance exercise showed a minimum $T_a$ of 13.4, 6.3, 2.8, 6.6°C, and maximum $T_a$ of 26.3, 19.0, 22.0, 18.8°C, respectively (HOBO data). The $T_a$ recorded by Bureau of Meteorology stations at varying distances from the event location are presented in **Table 1**.

## Individual GI Temperature, Heartrate and Speed Data During Endurance and Trotter Exercise Over Time

The individual $T_c$ parameters and descriptive analysis of $T_c$ are presented in **Supplementary Tables 1**, **3**. The descriptive analysis of speed, HR, H, and COT (endurance only) and duration of exercise of individual horses is presented in **Supplementary Table 2**.

## Overall $T_c$ Profiles During Endurance Exercise

The overall mean speed of endurance horses was 14.0 ± 1.4 km $h^{-1}$ over the first 40 km ($n$ = 11) and 14.2 ± 2.1 km $h^{-1}$ over the second 40 km ($n$ = 11) with a mean HR of 114 ± 13 bpm (**Supplementary Tables 1**, **2**). There was no significant correlation between end $T_c$ of the first 40 km endurance exercise loop and recovery time to HR ≤ 60 bpm and ($p$ = 0.646) but the end $T_c$ at the second 40 km showed a significant correlation with HR recovery ($p$ = 0.045). The cumulated H of the total 80 km was 120,000 ± 18,000 kJ. The $T_c$ profiles of endurance horses during exercise and recovery

[2]www.graphpad.com

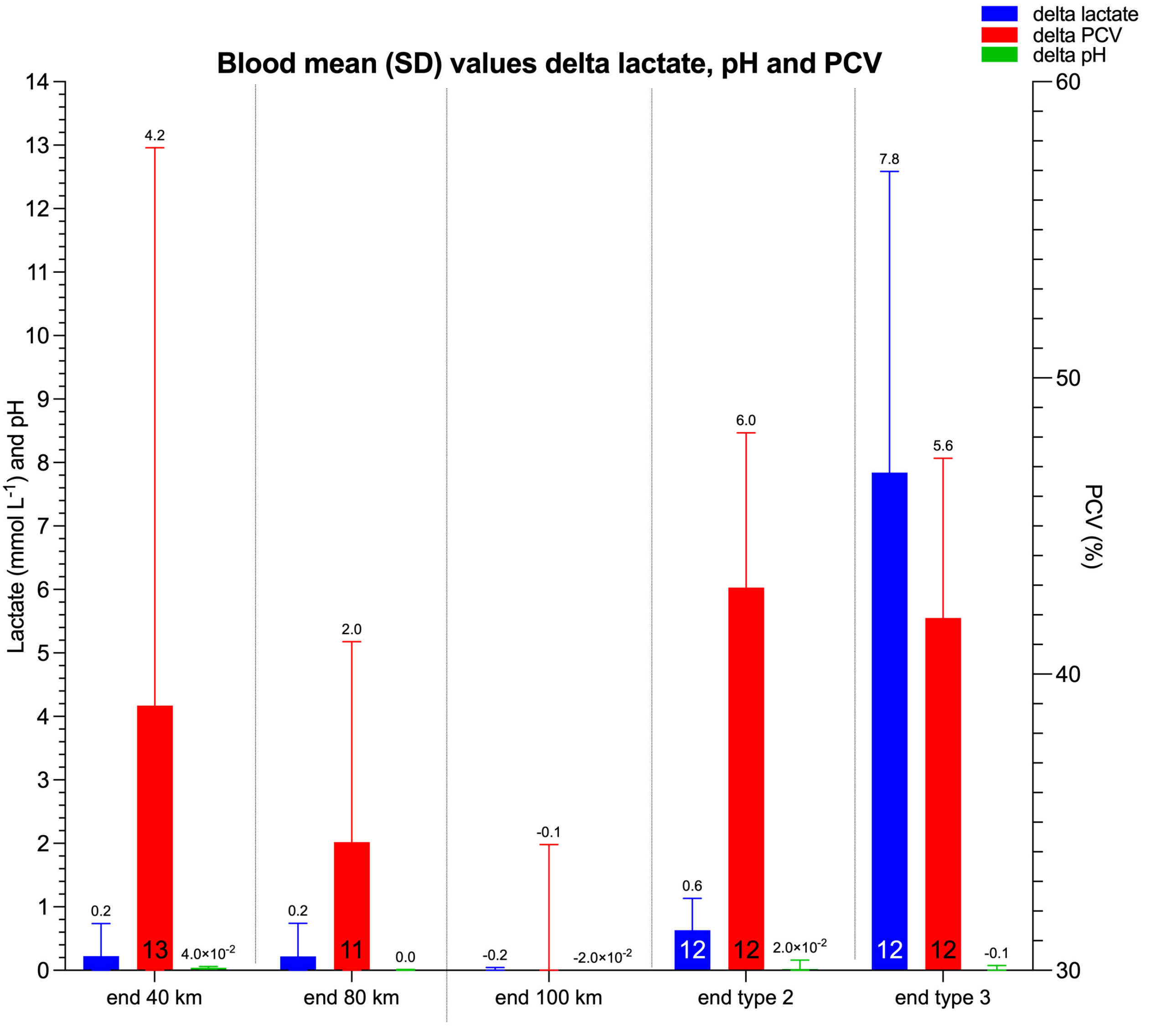


**FIGURE 7 |** Bar graph of blood parameters: lactate and pH on the *y*-axis presented as mmol $L^{-1}$ and the number, respectively, and the PCV (Hct) on the right y-axis presented as %. The type of exercise is presented on the *x*-axis as pre-, and post-ride in endurance and trotter exercises. The mean value is presented on top of the bar while sample size at the bottom in column.

are presented in **Table 2**, **Figures 3A**, **4**, and **Supplementary Tables 1**, **2**.

The mean max $T_c$, absolute max $T_c$, and net AUC of the first 40 km exercise were not significantly different from the second 40 km exercise [$F_{1,7} = 2.436$, $p = 0.163$ (for max $T_c$), $p = 0.66$ (for max absolute $T_c$), and $p = 0.56$ (for net AUC), respectively; **Figures 3A**, **6**]. Mean duration of all 40 km $T_c$ endurance exercise was 191 ± 43 min. The mean max $T_c$ during exercise was 39.0 ± 0.4°C and time to max $T_c$ was 143 ± 60 min at a mean distance of 29.5 ± 11.6 km. Hyperthermia was recorded in 50% of the $T_c$ exercise periods. The total dynamic thermal response (net AUC, $T_c$ °C × min) was 106.4 ± 44.3°C × min per 40 km and was approximately 0.5°C per min (**Figure 6**).

## Overall $T_c$ Profiles During Endurance Recovery

Post-exercise, only 25% of all $T_c$ recovery periods returned to their base $T_c$ while 50% decreased to or lower than 38°C within 39.6 ± 14 min. The time that $T_c$ returned to 38.5°C varied between 0 and 57 min (14/15 $T_c$ recovery periods). Horse 7 showed a different exercise $T_c$ time profile with an end of exercise $T_c$ of 39.4°C while the max $T_c$ (39.9°C) occurred at 57 min post-exercise (**Figure 4**). The dynamic thermal response during $T_c$ recovery period demonstrated a heat loss by a negative value (−18.1 ± 25.9°C × min) and a large variation amongst individuals (range 23.7–59.7°C × min) with a net AUC $min^{-1}$ −0.3 ± 0.4°C.

## Overall $T_c$ Profiles During Moderate Warm-Up Trotter Exercise and Recovery

The $T_c$ profiles of trotter horses during exercise and recovery are presented in **Table 2**, **Figures 3**, **5**, **Supplementary Tables 1**, **2**. The mean speed of the trotters during 1,540 m distance trotter exercise was 27.3 ± 5.3 km $h^{-1}$ with a mean HR of 140 ± 24 bpm (**Supplementary Table 2**). The $T_c$ profile of trotter moderate exercise revealed a minor $T_c$ increase and a minor dynamic thermal response during exercise and during recovery.

## Overall $T_c$ Profiles During Trotter Exercise

The mean speed of the trotters during 1,540 m distance trotter exercise was 40.0 ± 2.9 km $h^{-1}$ with a mean HR of 147 ± 17 bpm (**Supplementary Table 2**).

## Overall $T_c$ Profiles During Trotter Recovery

Post-exercise, HR recovered to 60 bpm in 5 of the 12 trotters and was coupled with a $T_c$ lower than 39.0°C in all those trotters. The $T_c$ profile of trotters reached their mean max $T_c$ of 38.8 ± 0.5°C during recovery within a mean time of 40.2 ± 30.2 min. The overall increase in mean $T_c$ (delta) varied (0.5 ± 0.5°C). Hyperthermia occurred in more than 40% of the trotters. Indeed, four trotters still had a $T_c$ higher than 39°C at the end of recovery, mean time of 68.6 ± 24.6 min duration. Only one $T_c$ returned to 38°C baseline during recovery. The $T_c$ profiles of trotter horses during exercise and recovery are presented in **Table 2**, **Figures 3**, **5**, and **Supplementary Table 1**. The dynamic thermal response was low during trotter exercise with a mean of 0.2 ± 0.4°C. However, thermoregulation in trotters occurred during recovery with a mean dynamic thermal response of 18.6 ± 22.1°C × min with a wide range (**Figure 6**) and 0.5 ± 0.3°C net AUC $min^{-1}$ duration of recovery (**Supplementary Table 3**).

## Comparison of the $T_c$ Profiles During Endurance and Trotter Exercise

The mean overall values of all data during the two different types of exercise are presented in **Supplementary Table 1**. The max $T_c$ in endurance exercise did not significantly differ from max $T_c$ in trotter exercise ($p$ = 0.19). In addition, the max $T_c$ and absolute max $T_c$ of the first 40 km and second 40 km endurance exercise loops did not significantly differ from trotter exercise when analyzed separately using an independent sample $t$-test ($p$ = 0.49 and $p$ = 0.26, respectively).

After 60 min recovery, hyperthermia persisted in only one endurance horse performing endurance exercise. This is in contrast to trotter exercise during which hyperthermia occurred during recovery in 5 of the 12 trotters.

## Blood Analysis Data in Endurance and Trotter Exercise

Blood results and the delta of lactate, pH, and Hct values of all horses (endurance and trotter exercise) are presented in **Figure 7**, **Supplementary Table 3**, and **Supplementary Figure 1**. An ANOVA revealed a significant difference in post-exercise delta lactate value between endurance (1.2 ± 0.6 mmol $L^{-1}$) and trotter (9.4 ± 4.0 mmol $L^{-1}$) as well as between warm-up trotter (1.6 ± 1.0 mmol $L^{-1}$) and fast trotter exercise with a higher lactate value in the last exercise [$F_{(2,38)}$ = 37.63, $p < 0.0001$]. No significant difference was identified between first and second 40 km endurance exercise ($p$ = 0.984), however, comparing both first and second 40 km endurance horses to trotter horses separately confirmed a highly significant difference ($p < 0.001$). In addition, mean delta lactate in the 40 km endurance exercise was significantly different to trotter exercise using an independent samples $t$-test ($p < 0.001$).

The delta Hct and delta pH values did not reveal significant differences between endurance and trotter exercise ($p$ = 0.60 and $p$ = 0.26, respectively).

# DISCUSSION

This is the first study to be conducted using a telemetric GI pill in field competitions to compare the equine thermoregulatory response to different types of exercise under normal weather conditions during the cooler South Australian months. For this purpose, a large dataset of $T_c$ recordings and other physiological responses was analyzed and compared between two types of exercise that differed greatly in duration and intensity (endurance compared with trotter exercise). As a result, an integrative real time view was obtained on how the equine body copes with heat load production during different types of exercise in the field. The GI pill proved to be a reliable tool to continuously monitor individual thermoregulatory well-being in sport horses.

There was an important difference in the time profile of the thermoregulatory response between endurance and trotter horses. Endurance horses reached their mean max $T_c$ during exercise toward the end of each leg of 40 km (after ± 30 km) and almost all returned to a $T_c$ lower than 38.5°C during 60 min into recovery. Trotter horses, however, always reached their mean max $T_c$ post-exercise at least 30 min into recovery. Moreover, in a third of the trotters, $T_c$ was still higher than 39°C and HR was higher than 60 bpm at the end of recovery. When HR is at 60 bpm, horses may still have a high $T_c$ (38.8 ± 0.4°C, range 38.0–39.7°C). Continuous monitoring of $T_c$ by a telemetric temperature-sensitive pill that passes through the GI tract has been evaluated in horses previously during rest and 158 km transport using the CorTemp® system (Green et al., 2005, 2008). While the Equivital system was validated in our previous study similarly during rest periods (Verdegaal et al., 2017), we applied this monitoring method in equine athletes specifically for the first time during field exercise.

The current study showed that the dynamic thermal response calculated by the net AUC proved to be a more useful presentation of the metabolic response rate than the calculated H (based on calculated values). Despite the individual variation of the max $T_c$ in endurance and trotter horses, it is of interest that the overall max $T_c$ in endurance horses (39.0 ± 0.4°C; range 38.5–39.9°C) was not significantly different to the max $T_c$ in trotters

(38.8 ± 0.5°C; range 37.6–39.3°C). Another important finding was that persisting hyperthermia was recorded in four trotters after a mean of 69 min post-exercise while only one endurance horse recorded 39.9°C after 60 min of recovery.

Most importantly, the current study has demonstrated that horses show important individual differences in their thermoregulatory response to different types of exercise. This result was obtained due to the continuous monitoring of the $T_c$ instead of performing serial measurements of $T_{re}$. For example, there was a large variation (range 11–40 km) between endurance horses with respect to the distance at which max $T_c$ was reached. It is noted that while the moment-to-moment location of the GI pill is unknown, based on a previously performed study we can state that $T_c$ changes are mainly attributed to exercise and less likely to the movement of the GI pill along the GI tract (Verdegaal et al., 2017). Both the individual variation in max $T_c$ and dynamic thermal response underline that individual continuous $T_c$ monitoring is essential for reliable oversight. This reliability is especially important since many horse-specific factors contribute to the thermoregulatory response, such as the genetic make-up of a horse, its temperament and its basic performance capacity level. However, for endurance horses, their performance capacity levels should have been quite similar for all studied horses, keeping in mind their similar competition experience levels for the 80 km distance (**Table 1**).

## Continuous Individual Monitoring Studies of the Thermoregulatory Response to Date

Based on recent equine and human studies, it is important to review different tools that allow for continuous reliable monitoring of the thermoregulatory response to exercise in the field with a minimal data loss. In that respect, the GI pill has proven to be a practical, accurate, and precise tool to monitor thermal response during field exercise both in human and equine athletes (Edwards and Clark, 2006; Verdegaal et al., 2017; Olcina et al., 2019).

A recent alternative method is the use of intra-muscular microchips embedded in different locations to continuously record muscle temperature (Kang et al., 2020). Although the study was performed under laboratory conditions, this technique could eventually be employed in the field. There was a positive correlation between muscle temperature and central venous temperature (CVT) only when combining all exercise phases including recovery. Interestingly during the recovery phase, the muscle temperature continued increasing and lagged behind the CVT. This non-alignment is in accordance with previous studies reporting on the lack of direct correlation between muscle temperature and core temperature (Byrd et al., 1989; McCutcheon et al., 1992; Hodgson et al., 1994). Therefore, more research is needed to obtain a better understanding of the associations between muscle temperature and core body temperature. One possible disadvantage of the technique is the requirement of invasive surgery to place the microchip.

## Comparison of the $T_c$ Profiles During Endurance and Trotter Exercise

The results of the current study are important for formulation of future competition and hot weather policies (MOOC endurance, 2021; MOOC heat illness, 2021). The study results show that both endurance and trotter horses reached comparable max $T_c$, although at different time points. Endurance horses reached their mean max $T_c$ (39.0 ± 0.3°C, 38.5–39.5°C) during endurance exercise toward the end of each leg of 40 km (after ± 30 km) and almost all $T_c$ returned to lower than 38.5°C during 60 min into recovery. In contrast, after the completion of trotter exercise, trotter horses reached their max $T_c$ (38.8 ± 0.5°C, 37.6–39.3°C) at an average of 34 min post-exercise with a mean $T_c$ increase of 0.6 ± 0.4°C (0–1.3°C). Moreover, in a third of the trotters, $T_c$ was still higher than 39°C and HR was higher than 60 bpm at the end of recovery. We found that within the 60-min rest time, all endurance horses achieved an overall recovery to 38.5°C in 15 out of 16 $T_c$ 40 km exercise periods, and 50% of these horses reached lower than 38°C. This supports the mandatory rest periods of 60 min as determined in the AERA and FEI endurance regulations when exercising in a cool environment (FEI, 2018; AERA, 2020). The present study was not able to demonstrate a correlation between the end $T_c$ first 40 km and recovery time to HR of 60 bpm but the end $T_c$ second 40 km was related to HR recovery. According to the AERA competition regulations, horses with a HR of 60 bpm at the end of their recovery period would meet all criteria to be labeled as "fit to continue" the competition while interestingly, we found the mean overall 40 km $T_c$ was still higher than normal (38.8 ± 0.4°C; range 38.0–39.7°C). This means that at the time that the HR recovered to 60 bpm, most horses were still actively coping with increased core body temperature. FEI regulations are stricter than AERA rules regarding the duration of the HR recovery time and require the HR to return below 64 bpm within 15 min post-exercise (FEI, 2021). Our study demonstrated that in the majority of $T_c$ periods (20 out of 22, $T_c$ 40 km exercise periods) the HR recovered to below 64 bpm within 15 min but again, the mean $T_c$ was still 38.7 ± 0.4°C (range 38.0–39.6°C; FEI, 2021). The HR recovery regulations in endurance horses are based on studies demonstrating that not only increased speed and distance but also a longer than 11–13 min cardiac recovery time is associated with higher risk of metabolic elimination after endurance exercise, more specifically with a 70% probability of elimination at the next veterinary check (Nagy et al., 2010; Younes et al., 2015; Bennet and Parkin, 2018). More field research is needed to investigate when heat accumulation in such horses can become problematic during follow-up exercise. The difference in thermoregulatory responses between endurance exercise and racing exercise is well-known and the current study provides exercise-specific core thermal response features which need to be addressed when formulating future equine competition and hot weather policies.

## Hyperthermia Post-exercise

Overall, 50% of the endurance horses developed hyperthermia during the standardized exercise loops, which underlines the

importance of an effective $T_c$ recovery period of sufficient duration. The persistence of hyperthermia in exercising endurance horses is one of the several factors causing metabolic disorders and fatigue due to the decreased supply of blood and fuel to the exercising muscles, brain and GI tract, and increased muscle and brain temperature (McCutcheon et al., 1992; Hodgson et al., 1993; Jones and Carlson, 1995; Geor and McCutcheon, 1998; Gonzalez-Alonso et al., 1999; McConaghy et al., 2002; Gerard et al., 2013; Brownlow et al., 2016; Brownlow and Mizzi, 2021).

While hyperthermia and heat stress are more commonly associated with prolonged exercise, we have demonstrated that horses performing short bouts of strenuous exercise in the field may suffer increasing from hyperthermia during recovery (1/3 of the trotters, **Figures 3B**, **5** and **Supplementary Table 1**).

Hyperthermia in racehorses post-exercise can be explained by the greater amount of metabolic heat generated by their larger muscle mass (compared to endurance horses) during their shorter exercise duration but higher intensity exercise (Hodgson et al., 1994; Geor et al., 1995; Jones and Carlson, 1995; Schott et al., 1999). In addition, the high intensity trotter exercise represents a short but acutely pronounced challenge to a multitude of organ systems at the same time such as the respiratory, cardiovascular, musculoskeletal and sympathetic nervous systems, of which the latter may be one of the driving forces behind the hyperthermia (Hetem et al., 2013). The "acuteness" may also explain the hyperthermia that was encountered in more trotter horses compared to endurance horses as well as the more significant interindividual variation seen at the level of the thermoregulatory response in trotter horses. Finally, the current study results underline the importance for formulating guidelines for the management and welfare of all equine sports disciplines. These physiological factors, coupled with environmental factors in the field, can overwhelm the capacity of the heat loss mechanisms of sport horses. It is important to point out that there is no scientific evidence to date suggesting that horses experience heat injury when their $T_c$ reaches and is sustained at 39°C. However, it should be kept in mind that if horses, and especially trotters, engage in a subsequent exercise bout too soon, their core body temperatures would be starting at an elevated level.

In most trotters (7/12), the first signs toward lowering and normalizing core body temperature became visible after a minimum of 20 min into the recovery. Problems may arise when the hyperthermia in racehorses (with or without clinical signs) is not recognized. The delayed onset of hyperthermia may go unnoticed when trotters are transported shortly after completion of the race. Hyperthermia coupled with transport during hot weather is likely to increase heat loading. The identification of ongoing hyperthermia during recovery is essential information with respect to equine welfare and emphasizes the need for extended continuous monitoring post-exercise. Our findings may explain personal anecdotal evidence expressed by trotter trainers noticing that some horses show discomfort the day after an otherwise uneventful competition day (Courouce et al., 2002; Richard et al., 2010; Bertuglia et al., 2014) due to unrecognized post-racing hyperthermia (McConaghy et al., 2002; Brownlow and Mizzi, 2020, 2021). The current findings also underline that the habit of withholding water prior to racing events is not an ideal practice. Indeed, several studies involving endurance or trotter horses report that dehydration and electrolyte loss are significant predictors for early elimination due to metabolic reasons (Barton et al., 2003; Fielding et al., 2009; Barnes et al., 2010; Munoz et al., 2010; Trigo et al., 2010; Waller and Lindinger, 2010). Likewise, a recent meta-analysis in human athletes demonstrated the importance of fluid ingestion to counteract hyperthermia and to improve performance capacity (Alhadad et al., 2019). In our study, no fluid intake restrictions were applied and blood values showed that the studied horses were not dehydrated.

As in endurance horses, the recovery for trotters to HR 60 bpm was not associated with a recovered $T_c$. This is important knowledge for the monitoring of trotters during recovery when using HR as guidance. At a HR of 60 bpm point in time, the mean $T_c$ was still 38.7 ± 0.5°C (range 38.0–39.3°C) while 8/12 trotters still measured a $T_c$ higher than 38.5°C. A post-exercise peak $T_{re}$ was earlier reported in a few other studies (Hodgson et al., 1993; Kohn and Hinchcliff, 1995; Marlin et al., 1996, 1998; Kohn et al., 1999; Foreman et al., 2006; Verdegaal et al., 2017) and supports the conclusion that it is necessary to continue monitoring racehorses during recovery. Based on our findings and coupled with those reported by previous studies, continuous monitoring of racehorses and sport horses for hyperthermia using the GI pill or $T_{re}$ during a period of rest for at least 60 min post-exercise is highly recommended.

## The Dynamic Thermal Response (Net AUC $T_c$)

The continuous $T_c$ monitoring allowed for the construction of detailed temperature-time profiles and the subsequent application of the AUC approach. The present study measured net AUC $T_c$ to assess the dynamic thermal response to the metabolic thermal load, a product of the thermoregulatory mechanisms that increase temperature over time (Hodgson et al., 1993; Schroter and Marlin, 1995; Schott et al., 1999; Hodgson, 2014). As expected, our study demonstrated that the mean dynamic thermal response was much higher during endurance exercise compared to trotters due to higher H over time. The core thermal response during recovery in endurance horses was negative due to metabolic heat dissipation. Alternatively in trotters, both heat load and heat loss mechanisms were in place during recovery, represented by a relatively high dynamic thermal response compared to the short duration of the exercise. The thermal response index using area under the fever curve has been applied in several mammal studies comparing the fever response over time (Feng and Lipton, 1987; Murakami and Ono, 1987). Only a few equine research groups report the use of AUC to assess temperature response over time. One equine study used AUC to demonstrate changes in muscle and tendon tissue temperature over time during therapeutic ultrasound treatments at different tissue depths (Montgomery et al., 2013). Studies involving human athletes have used AUC to compare AUC temperature response in two study groups. One study used AUC

to quantify the body temperature response over time (60 min cycle exercise at 65% $\dot{V}O_2max$) by continuous recording of $T_{re}$ and demonstrated that $T_{re}$ AUC response was not different between ingesting a carbohydrate drink [47.7 ± 11.6 (°C × min)] and a placebo [50.1 ± 11.1 (°C × min)]—unfortunately recovery data were not reported (Horswill et al., 2008). Another study reviewed comparisons of different cooling methods using AUC ($T_{re}$ to time) to assess cooling rates over time and argued that a larger AUC was associated with an increased risk of tissue and organ injury (Casa et al., 2007). A recent human study used AUC to quantify the thermal response during bladder cancer treatment to assess its use as a prognostic parameter and concluded AUC was a simple calculation to predict the medical outcome (Datta et al., 2021). Similarly, we can argue that in our study a high net AUC (high dynamic thermal response) may be associated with a higher risk of thermally based injury due to insufficient thermoregulatory mechanisms. When comparing the values of the calculated H and calculated COT in our study across endurance exercise (according to Schroter and Marlin, 2002), the H calculations were not discriminative for endurance horses with high max $T_c$. Moreover, these calculations did not consider anaerobic exercise and therefore could not be applied to trotter exercise for comparison. Another concern is that the H equation is formula-based using several calculated values such as $\dot{V}O_2$. When comparing this calculated H method to the AUC method which can only be used in association with a continuous monitoring technique, the latter seems a much more accurate approach.

## Blood Values in Response to Exercise

Blood lactate values showed only a mild increase in endurance horses and were not different between the first and second 40 km endurance exercise. These values were similar to an earlier report which identified no difference in blood lactate values between eliminated endurance horses and finishers (Fielding et al., 2009).

# LIMITATIONS

Our current study has various limitations that should be considered when assessing our findings. As applies in all field studies, not all conditions could be 100% controlled, such as perfectly equal conditions of competition tracks, $T_a$, solar radiation, and the fact that the endurance part involved privately owned horses which obviously received different diets and followed different trainings protocols. In retrospect, scraping off water from the horses during cooling down was not the most optimal approach since recent research comparing five cooling methods in racehorses favored continuous reapplication of cold water without subsequently scraping it off (Takahashi et al., 2020). We did not seek to investigate individual external horse-related influencing factors such as diet, supplements, health history, duration of transport prior to competition nor processes such as the circadian rhythms which may have caused a diurnal variation in $T_c$. Future research is needed to investigate these variables to augment our understanding of heat load production in sport horses in varying ambient conditions.

# CONCLUSION

Continuous monitoring of $T_c$ using the GI telemetry pill is a reliable, non-invasive method of assessing the thermoregulatory response in exercising horses. It provides vital individual recording and monitoring of important interindividual differences in $T_c$ time profiles. Clearly, seeking a universal thermoregulatory response pattern that will apply to all exercising horses will prove futile. Consequently, continuous monitoring allows for applying an AUC approach which is a more robust parameter when compared to calculated H. In our study, endurance horses reached their max $T_c$ on average when completing over 75% of a 40 km exercise leg; this returned to baseline within the mandatory 60 min recovery time. However, several horses still had $T_c$ values above 38.5°C when their HR had already returned to 60 bpm which is used in endurance (AERA) as "fit to continue" competing. Trotter horses reached their peak $T_c$ during their recovery period on average at 34 min after exercise. Here again, several horses had $T_c$ values above 38.5°C with a HR already returned to 60 bpm. Our results have shown that $T_c$ monitoring should continue in trotter horses at least until 60 min post-exercise.

Future research involving strenuously exercising horses should focus on the possible impact of intrinsic characteristics such as genetics and training, as well as more challenging environmental conditions. Our findings have implications for improving the overall welfare of sport horses and future management of equine welfare at sport events such as the Olympic Games (Hosokawa et al., 2020; Elliott, 2021). Our current study provides reliable supporting evidence for the need for industry-wide, temperature-monitoring guidelines to prevent EHI in endurance horses and racehorses when exercising and recovering in the field.

# DATA AVAILABILITY STATEMENT

The original contributions presented in the study are included in the article/**Supplementary Material**, further inquiries can be directed to the corresponding author/s.

# ETHICS STATEMENT

The animal study was reviewed and approved by University of Adelaide Animal Ethics Committee (project number S-2011-224) Australia. Written informed consent was obtained from the owners for the participation of their animals in this study.

# AUTHOR CONTRIBUTIONS

E-LV developed the study design and contributed to data collection, preparation, and creation of the database, descriptive and statistical analysis, interpretation of the data, and writing of the manuscript. CD was involved in study design, preparation, and creation of the database, analysis and interpretation of the data, and writing of the manuscript. GH contributed to

drafting and revision of the manuscript. SJ was involved in study design and data collection. TM contributed to reviewing the manuscript and statistical analysis. BB and CV were involved in data file setup and reviewing the manuscript. SF was involved in the initial design and contributed to data collection. LF and SF were involved in the initial design of the study and data collection for the trotter horses. All authors read and approved the final manuscript.

## FUNDING

This study was supported by REM Systems, Australia through the supply of blood analysis equipment; and financially supported by a grant of $24,000 from the University of Adelaide for the cost of monitoring equipment.

## ACKNOWLEDGMENTS

The authors are deeply grateful to all the participating endurance horse owners and the SAERA organization for their welcome logistic support. The authors would like to acknowledge Louise Niva for her excellent editorial assistance, Jeroen Dewulf and Charles G. B. Caraguel for their statistical advice and Ben Greenaway (BMedical, Australia) for his technical support.

## SUPPLEMENTARY MATERIAL

The Supplementary Material for this article can be found online at: https://www.frontiersin.org/articles/10.3389/fphys.2021.708737/full#supplementary-material

**Supplementary Figure 1 |** Bar graph comparing delta blood parameters lactate, pH and PCV (Hct) pre-, and post-ride in endurance and trotter exercise.

**Supplementary Table 1 |** $T_c$ parameters in endurance exercise (per 40 km) and trotter exercise.

**Supplementary Table 2 |** Global Positioning System (GPS), heartrate (HR) values and calculated values related to metabolic heat production (H).

**Supplementary Table 3 |** Blood parameters of endurance horses and trotters (only lactate, PCV, pH) pre- and post-ride.

## REFERENCES


Adams, W. M., and Jardine, J. F. (2020). "Overview of exertional heat illness," in *Exertional heat illness*, eds W. M. Adams and J. F. Jardine (Berlin: Springer), 1–16. doi: 10.1007/978-3-030-27805-2_1

AERA (2020). *A.E.R.A. Rulebook*. Marawa PI: Australian Endurance Riding Association.

Alhadad, S. B., Tan, P. M. S., and Lee, J. K. W. (2019). Efficacy of heat mitigation strategies on core temperature and endurance exercise: A meta-analysis. *Front. Physiol.* 10:71. doi: 10.3389/fphys.2019.00071

Averay, K., Nicholson, A., and Verdegaal, E. J. M. M. (2014). "Preliminary evaluation of the veterinary Epoc® point-of-care (poc) clinical analyser in horses by comparison with in-house analysis and establishment of reference values," in *13th Scientific Conference of European Vet. Emerg. & Crit. Soc. (EVECCS)*, (Prague: J. Vet. Emerg. Crit. Care).

Barnes, A., Kingston, J., Beetson, S., and Kuiper, C. (2010). Endurance veterinarians detect physiologically compromised horses in a 160 km ride. *Equine Vet. J. Suppl.* 2010, 6–11. doi: 10.1111/j.2042-3306.2010.00225

Barton, M. H., Williamson, L., Jacks, S., and Norton, N. (2003). Body weight, hematologic findings, and serum and plasma biochemical findings of horses competing in a 48-, 83-, or 159-km endurance ride under similar terrain and weather conditions. *Am. J. Vet. Res.* 64, 746–753. doi: 10.2460/ajvr.2003.64.746

Bennet, E. D., and Parkin, T. D. H. (2018). Federation Equestre Internationale endurance events: Risk factors for failure to qualify outcomes at the level of the horse, ride and rider (2010-2015). *Vet. J.* 236, 44–48. doi: 10.1016/j.tvjl.2018.04.011

Bertuglia, A., Bullone, M., Rossotto, F., and Gasparini, M. (2014). Epidemiology of musculoskeletal injuries in a population of harness standardbred racehorses in training. *BMC Vet. Res.* 10:11. doi: 10.1186/1746-6148-10-11

BOM (2020). *BOM*. Melbourne: Bureau for Meteorology. Available online at: http://www.bom.gov.au/climate/data/ (accessed December 2, 2020).

Brownlow, M. A., and Mizzi, J. X. (2020). Thermoregulatory capacity of the thoroughbred racehorse and its relationship to the pathogenesis of exertional heat illness. *Equine Vet. Ed.* 8:13433. doi: 10.1111/eve.13433

Brownlow, M. A., Dart, A. J., and Jeffcott, L. B. (2016). Exertional heat illness: A review of the syndrome affecting racing thoroughbreds in hot and humid climates. *Aus. Vet. J.* 94, 240–247. doi: 10.1111/avj.12454

Brownlow, M., and Mizzi, J. (2021). Exertional heat illness in thoroughbred racehorses – pathophysiology, case definition and treatment rationale. *Equine Vet. Ed.* 2021:13459. doi: 10.1111/eve.13459

Byrd, S. K., Mccutcheon, L. J., Hodgson, D. R., and Gollnick, P. D. (1989). Altered sarcoplasmic reticulum function after high-intensity exercise. *J. Appl. Physiol.* 67, 2072–2077. doi: 10.1152/jappl.1989.67.5.2072

Casa, D. J., Mcdermott, B. P., Lee, E. C., Yeargin, S. W., Armstrong, L. E., and Maresh, C. M. (2007). Cold water immersion: The gold standard for exertional heatstroke treatment. *Exerc. Sport Sci. Rev.* 35, 141–149. doi: 10.1097/jes.0b013e3180a02bec

Courouce, A., Chretien, M., and Valette, J. P. (2002). Physiological variables measured under field conditions according to age and state of training in French Trotters. *Equine Vet. J.* 34, 91–97. doi: 10.2746/042516402776181141

Courouce-Malblanc, A. (2013). *Clinical exercise testing*, Chap. 29. Amsterdam: Elsevier Health Sciences.

Datta, N. R., Marder, D., Datta, S., Meister, A., Puric, E., Stutz, E., et al. (2021). Quantification of thermal dose in moderate clinical hyperthermia with radiotherapy: A relook using temperature-time area under the curve (auc). *Int. J. Hyperthermia* 38, 296–307. doi: 10.1080/02656736.2021.1875060

Edwards, A. M., and Clark, N. A. (2006). Thermoregulatory observations in soccer match play: Professional and recreational level applications using an intestinal pill system to measure core temperature. *Br. J. Sports Med.* 40, 133–138. doi: 10.1136/bjsm 2005.021980

Elliott, C. (2021). Clinical insights: Preparing for the Tokyo Olympics-ensuring health and welfare of equine athletes in the face of heat and humidity. *Equine Vet. J.* 2021:13446. doi: 10.1111/evj.13446

FEI (2018). *F.E.I. Veterinary regulations*, 14th Edn. Lausanne: International Federation for Equestrian Sports.

FEI (2021). *F.E.I. Endurance rules updated*, 11th Edn. Lausanne: International Federation for Equestrian Sports.

Feng, J., and Lipton, J. M. (1987). Eugenol- antipyretic activity in rabbits. *Neuropharm* 26, 1775–1778. doi: 10.1016/0028-3908(87)90131-6

Fielding, C. L., Magdesian, K. G., Rhodes, D. M., Meier, C. A., and Higgins, J. C. (2009). Clinical and biochemical abnormalities in endurance horses eliminated from competition for medical complications and requiring emergency medical treatment: 30 cases (2005-2006). *J. Vet. Emerg. Crit. Care* 19, 473–478. doi: 10.1111/j.1476-4431.2009.00441

Fielding, C. L., Meier, C. A., Balch, O. K., and Kass, P. H. (2011). Risk factors for the elimination of endurance horses from competition. *J. Am. Vet. Med. Assoc.* 239, 493–498. doi: 10.2460/javma.239.4.493

Fielding, C. L., Meier, C. A., Fellers, G. K., and Magdesian, K. G. (2017). Ability of clinicopathologic variables and clinical examination findings to predict race elimination in endurance horses. *Am. J. Vet. Res.* 78, 50–56. doi: 10.2460/ajvr.78.1.50

Foreman, J. H., Benson, G. J., and Foreman, M. H. (2006). Effects of a pre-moistened multilayered breathable fabric in promoting heat loss during recovery after exercise under hot conditions. *Equine Vet. J. Suppl.* 2006, 303–307. doi: 10.1111/j.2042-3306.2006.tb05558

Geor, R. J., and McCutcheon, L. J. (1998). Hydration effects on physiological strain of horses during exercise-heat stress. *J. Appl. Physiol.* 84, 2042–2051. doi: 10.1152/jappl.1998.84.6.2042

Geor, R. J., McCutcheon, L. J., Ecker, G. L., and Lindinger, M. I. (1995). Thermal and cardiorespiratory responses of horses to submaximal exercise under hot and humid conditions. *Equine Vet. J. Suppl.* 1995, 125–132. doi: 10.1111/j.2042-3306.1995.tb05018.x

Gerard, M. P., De Graaf-Roelfsema, E., Hodgson, D. R., and Van der Kolk, J. H. (2013). "Metabolic energetics," in *Equine Locomotion*, eds W. Back and H. M. Clayton (Amsterdam: Elsevier health sciences), 419–442.

Gonzalez-Alonso, J., Teller, C., Andersen, S. L., Jensen, F. B., Hyldig, T., and Nielsen, B. (1999). Influence of body temperature on the development of fatigue during prolonged exercise in the heat. *J. Appl. Physiol.* 86, 1032–1039. doi: 10.1152/jappl.1999.86.3.1032

Green, A. R., Gates, R. S., Lawrence, L. M., and Wheeler, E. F. (2008). Continuous recording reliability analysis of three monitoring systems for horse core body temperature. *Comput. Electron. Agric.* 61, 88–95. doi: 10.1016/j.compag.2007.09.012

Green, A. R., Gates, S. G., and Lawrence, L. M. (2005). Measurement of horse core body temperature. *J. Therm. Biol.* 30, 370–377. doi: 10.1016/j.jtherbio.2005.03.003

Hargreaves, B. J., Kronfeld, D. S., and Naylor, J. R. (1999). Ambient temperature and relative humidity influenced packed cell volume, total plasma protein and other variables in horses during an incremental submaximal field exercise test. *Equine Vet. J.* 31, 314–318. doi: 10.1111/j.2042-3306.1999.tb03823

Hetem, R. S., Mitchell, D., de Witt, B. A., Fick, L. G., Meyer, L. C., Maloney, S. K., et al. (2013). Cheetah do not abandon hunts because they overheat. *Biol. Lett.* 9:0472. doi: 10.1098/rsbl.2013.0472

Hodgson, D. R. (2014). "Thermoregulation," in *The athletic horse, principles and practice of equine sport medicine*, 2nd Edn, eds C. McGowan, D. R. Hodgson, and K. McKeever (St. Louis: Elsevier), </UBLOC>108–124.

Hodgson, D. R., Davis, R. E., and McConaghy, F. F. (1994). Thermoregulation in the horse in response to exercise. *Br. Vet. J.* 150, 219–235. doi: 10.1016/s0007-1935(05)80003-x

Hodgson, D. R., McCutcheon, L. J., Byrd, S. K., Brown, W. S., Bayly, W. M., Brengelmann, G. L., et al. (1993). Dissipation of metabolic heat in the horse during exercise. *J. Appl. Physiol.* 74, 1161–1170. doi: 10.1152/jappl.1993.74.3.1161

Horswill, C. A., Stofan, J. R., Lovett, S. C., and Hannasch, C. (2008). Core temperature and metabolic responses after carbohydrate intake during exercise at 30 degrees C. *J. Athl. Train.* 43, 585–591. doi: 10.4085/1062-6050-43.6.585

Hosokawa, Y., Casa, D. J., and Racinais, S. (2020). Translating evidence-based practice to clinical practice in Tokyo 2020: how to diagnose and manage exertional heat stroke. *Brit. J. Sports Med.* 54, 883–884. doi: 10.1136/bjsports-2020-102153

Hosokawa, Y., Nagata, T., and Hasegawa, M. (2019). Inconsistency in the standard of care-toward evidence-based management of exertional heat stroke. *Front. Physiol.* 10:108. doi: 10.3389/fphys.2019.00108

IUPS Thermal Commission (2001). Glossary of terms for thermal physiology. *Jpn. J. Physiol.* 51, 245–280.

Jeffcott, L., Leung, W. M., and Riggs, C. (2009). Managing the effects of the weather on the equestrian events of the 2008 Beijing Olympic games. *Vet. J.* 182, 412–429. doi: 10.1016/j.tvjl.2009.07.037

Jones, J. H., and Carlson, G. P. (1995). Estimation of metabolic energy cost and heat production during a 3-day-event. *Equine Vet. J. Suppl.* 1995, 23–30. doi: 10.1111/j.2042-3306.1995.tb05004

Kang, H., Zsoldos, R. R., Woldeyohannes, S. M., Gaughan, J. B., and Sole Guitart, A. (2020). The use of percutaneous thermal sensing microchips for body temperature measurements in horses prior to, during and after treadmill exercise. *Animals* 2020:ani10122274. doi: 10.3390/ani10122274

Kirsch, K., Detilleux, J., Serteyn, D., and Sandersen, C. (2019). Comparison of two portable clinical analyzers to one stationary analyzer for the determination of blood gas partial pressures and blood electrolyte concentrations in horses. *PLoS One* 14:e0211104. doi: 10.1371/journal.pone.0211104

Kohn, C. W., and Hinchcliff, K. W. (1995). Physiological responses to the endurance test of a 3-day-event during hot and cool weather. *Equine Vet. J. Suppl.* 1995, 31–36. doi: 10.1111/j.2042-3306.1995.tb05005

Kohn, C. W., Hinchcliff, K. W., and Mckeever, K. H. (1999). Effect of ambient temperature and humidity on pulmonary artery temperature of exercising horses. *Equine Vet. J. Suppl.* 1999, 404–411. doi: 10.1111/j.2042-3306.1999.tb05256

Kohn, C. W., Hinchcliff, K. W., McCutcheon, L. J., Geor, R., Foreman, J., Allen, A. K., et al. (1995). Physiological responses of horses competing at a modified 1 star 3-day-event. *Equine Vet. J. Suppl.* 1995, 97–104. doi: 10.1111/j.2042-3306.1995.tb05014

Legg, K. A., Weston, J. F., Gee, E. K., Bolwell, C. F., Bridges, J. P., and Rogers, C. W. (2019). Characteristics of endurance competitions and risk factors for elimination in New Zealand during six seasons of competition (2010/11-2015/16). *Animals* 9:ani9090611. doi: 10.3390/ani9090611

Marlin, D. J., Scott, C. M., Roberts, C. A., Casas, I., Holah, G., and Schroter, R. C. (1998). Post exercise changes in compartmental body temperature accompanying intermittent cold water cooling in the hyperthermic horse. *Equine Vet. J.* 30, 28–34. doi: 10.1111/j.2042-3306.1998.tb04085

Marlin, D. J., Scott, C. M., Schroter, R. C., Mills, P. C., Harris, R. C., Harris, P. A., et al. (1996). Physiological responses in nonheat acclimated horses performing treadmill exercise in cool (20 degrees C/40% RH), hot dry (30 degrees C/40% RH) and hot humid (30 degrees C/80% rh) conditions. *Equine Vet. J. Suppl.* 1996, 70–84. doi: 10.1111/j.2042-3306.1996.tb05034.x

McConaghy, F. F., Hodgson, D. R., Hales, J. R., and Rose, R. J. (2002). Thermoregulatory-induced compromise of muscle blood flow in ponies during intense exercise in the heat: A contributor to the onset of fatigue? *Equine Vet. J. Suppl.* 2002, 491–495. doi: 10.1111/j.2042-3306.2002.tb05471

McCutcheon, L. J., and Geor, R. J. (2008). "Thermoregulation and exercise-associated heat stress," in *Equine Exerc. Physiol., The science of exercise in the athletic horse*, eds K. W. Hinchcliff, R. J. Geor, and A. J. Kaneps (Philadelphia: Saunders Ltd.), 382–396. doi: 10.1016/b978-070202857-1.50018-4

McCutcheon, L. J., Byrd, S. K., and Hodgson, D. R. (1992). Ultrastructural-changes in skeletal-muscle after fatiguing exercise. *J. Appl. Physiol.* 72, 1111–1117. doi: 10.1152/jappl.1992.72.3.1111

Misheff, M. M. (2020). "Metabolic disorders in endurance horses," in *Blackwell's five-minute Veterinary consult: Equine*, ed. J. Lavoie (New Jersey, NY: Wiley Blackwell), 485–486.

Mitchell, G., Fuller, A., Maloney, S. K., Rump, N., and Mitchell, D. (2006). Guttural pouches, brain temperature and exercise in horses. *Biol. Lett.* 2, 475–477. doi: 10.1098/rsbl.2006.0469

Montgomery, L., Elliott, S. B., and Adair, H. S. (2013). Muscle and tendon heating rates with therapeutic ultrasound in horses. *Vet. Surg.* 42, 243–249. doi: 10.1111/j.1532-950X.2013.01099

MOOC endurance (2021). *The science of endurance training and performance*. Cambridge: Harvard University.

MOOC heat illness (2021). *The health effects of climate change*. Cambridge: Harvard University.

Munoz, A., Riber, C., Trigo, P., Castejon-Riber, C., and Castejon, F. M. (2010). Dehydration, electrolyte imbalances and renin-angiotensin-aldosterone-vasopressin axis in successful and unsuccessful endurance horses. *Equine Vet. J. Suppl.* 2010, 83–90. doi: 10.1111/j.2042-3306.2010.00211

Murakami, N., and Ono, T. (1987). Sex-related differences in fever development of rats. *A. J. Phys. Reg. Integ. Comp. Phys.* 252, 284–289.

Nagy, A., Dyson, S. J., and Murray, J. K. (2012). A veterinary review of endurance riding as an international competitive sport. *Vet. J.* 194, 288–293. doi: 10.1016/j.tvjl.2012.06.022

Nagy, A., Murray, J. K., and Dyson, S. (2010). Elimination from elite endurance rides in nine countries: A preliminary study. *Equine Vet. J. Suppl.* 2010, 637–643. doi: 10.1111/j.2042-3306.2010.00220

Nagy, A., Murray, J. K., and Dyson, S. J. (2014). Descriptive epidemiology and risk factors for eliminations from Federation Equestre Internationale endurance

rides due to lameness and metabolic reasons (2008-2011). *Equine Vet. J.* 46, 38–44. doi: 10.1111/evj.12069

Olcina, G., Crespo, C., Timon, R., Mjaanes, J. M., and Calleja-Gonzalez, J. (2019). Core temperature response during the marathon portion of the ironman world championship (Kona-Hawaii). *Front. Physiol.* 10:1469. doi: 10.3389/fphys.2019.01469

Pagan, J. D., and Hintz, H. F. (1986). Equine energetics. I. Relationship between body weight and energy requirements in horses. *J. Anim. Sci.* 63, 815–821. doi: 10.2527/jas1986.633815x

Parker, M., Goodwin, D., Eager, R. A., Redhead, E. S., and Marlin, D. J. (2009). Comparison of Polar® heart rate interval data with simultaneously recorded ECG signals in horses. *Comp. Exerc. Phys.* 6, 137–142. doi: 10.1017/S1755254010000024

Raymond, C., Matthews, T., and Horton, R. M. (2020). The emergence of heat and humidity too severe for human tolerance. *Sci. Adv.* 6:aaw1838. doi: 10.1126/sciadv.aaw1838

Richard, E. A., Fortier, G. D., Pitel, P. H., Dupuis, M. C., Valette, J. P., Art, T., et al. (2010). Sub-clinical diseases affecting performance in standardbred trotters: Diagnostic methods and predictive parameters. *Vet. J.* 184, 282–289. doi: 10.1016/j.tvjl.2009.04.016

Schott, H. C. II, Dusterdieck, K. F., Eberhart, S. W., Woody, K. A., Refsal, K. R., and Coenen, M. (1999). Effects of electrolyte and glycerol supplementation on recovery from endurance exercise. *Equine Vet. J. Suppl.* 1999, 384–393. doi: 10.1111/j.2042-3306.1999.tb05253

Schroter, R. C., and Marlin, D. J. (1995). An index of the environmental thermal load imposed on exercising horses and riders by hot weather conditions. *Equine Vet. J. Suppl.* 1995, 16–22. doi: 10.1111/j.2042-3306.1995.tb05003

Schroter, R. C., and Marlin, D. J. (2002). Modelling the oxygen cost of transport in competitions over ground of variable slope. *Equine Vet. J. Suppl.* 2002, 397–401. doi: 10.1111/j.2042-3306.2002.tb05455

Smith, J. E., Barnes, A. L., and Maloney, S. K. (2006). A nonsurgical method allowing continuous core temperature monitoring in mares for extended periods, including during endurance exercise. *Equine Vet. J. Suppl.* 2006, 65–69. doi: 10.1111/j.2042-3306.2006.tb05515.x

Takahashi, Y., and Takahashi, T. (2020). Risk factors for exertional heat illness in thoroughbred racehorses in flat races in japan (2005-2016). *Equine Vet. J.* 52, 364–368. doi: 10.1111/evj.13179

Takahashi, Y., Ohmura, H., Mukai, K., Shiose, T., and Takahashi, T. (2020). A comparison of five cooling methods in hot and humid environments in thoroughbred horses. *J. Equine Vet. Sci.* 91:103130. doi: 10.1016/j.jevs.2020.103130

Trigo, P., Castejon, F., Riber, C., and Munoz, A. (2010). Use of biochemical parameters to predict metabolic elimination in endurance rides. *Equine Vet. J. Suppl.* 2010, 142–146. doi: 10.1111/j.2042-3306.2010.00238

Verdegaal, E. J. M. M., Delesalle, C., Caraguel, C. G. B., Folwell, L. E., McWhorter, T. J., Howarth, G. S., et al. (2017). Evaluation of a telemetric gastrointestinal pill for continuous monitoring of gastrointestinal temperature in horses at rest and during exercise. *Am. J. Vet. Res.* 78, 778–784. doi: 10.2460/ajvr.78.7.778

Verdegaal, E. J. M. M., Delesalle, C., McWorther, T. J., Howarth, G. S., Mcleod, K., Weaver, S., et al. (2018). "Incidence and description of exertional rhabdomyolysis in endurance horses at 160 km cup 2017," in *10th Int. Conf. Equine Exerc. Physiol. (ICEEP)*, (Lorne: *ICEEP*), 14.

Wagner, E. L., and Tyler, P. J. (2011). A comparison of weight estimation methods in adult horses. *J. Equine Vet. Sc.* 31, 706–710. doi: 10.1016/j.jevs.2011.05.002

Waller, A. P., and Lindinger, M. I. (2010). Nutritional aspects of post exercise skeletal muscle glycogen synthesis in horses: A comparative review. *Equine Vet. J.* 42, 274–281. doi: 10.2746/042516409X479603

Wallsten, H., Olsson, K., and Dahlborn, K. (2012). Temperature regulation in horses during exercise and recovery in a cool environment. *Acta Vet. Scand.* 54:42. doi: 10.1186/1751-0147-54-42

Younes, M., Robert, C., Cottin, F., and Barrey, E. (2015). Speed and cardiac recovery variables predict the probability of elimination in equine endurance events. *PLoS One* 10:e0137013. doi: 10.1371/journal.pone.0137013

**Conflict of Interest:** The authors declare that the research was conducted in the absence of any commercial or financial relationships that could be construed as a potential conflict of interest.

**Publisher's Note:** All claims expressed in this article are solely those of the authors and do not necessarily represent those of their affiliated organizations, or those of the publisher, the editors and the reviewers. Any product that may be evaluated in this article, or claim that may be made by its manufacturer, is not guaranteed or endorsed by the publisher.

*Copyright © 2021 Verdegaal, Howarth, McWhorter, Boshuizen, Franklin, Vidal Moreno de Vega, Jonas, Folwell and Delesalle. This is an open-access article distributed under the terms of the Creative Commons Attribution License (CC BY). The use, distribution or reproduction in other forums is permitted, provided the original author(s) and the copyright owner(s) are credited and that the original publication in this journal is cited, in accordance with accepted academic practice. No use, distribution or reproduction is permitted which does not comply with these terms.*
