# Supplementary material for: Is Continuous Monitoring of Skin Surface Temperature a Reliable Proxy to Assess the Thermoregulatory Response in Endurance Horses During Field Exercise?
Source: Front Vet Sci. 2022 May 27;9:894146. doi: 10.3389/fvets.2022.894146 (PMC9196037; doi:10.3389/fvets.2022.894146)
Supplement: Supplementary file 2 [file Table_1.docx]

**Supplementary table – Monitoring skin temperature in endurance horses**

**Summary of a total of 30 selected studies measuring the surface T*_sk_* in horses.**

- 11 studies at rest
- 19 studies related to exercise including:
  - Seven studies continuous monitoring using T*_sk_* **contact sensors**:
    - One field study using i-Button^®^ (Klous et al., 2020)
    - Five laboratory-based studies in the 1990’s: Marlin et al. (n = 3, using thermistor probe) & Geor et al. (n = 2, using thermocouple)
    - One recent study using dynamic infrared thermography (IRT) camera (Soroko et al., 2018)
  - 12 studies measured T*_sk_* post-exercise at a single point including one study with T*_sk_* both pre- and post-exercise

**Monitoring continuous or not continuous**

A total of nine studies monitored T*_sk_* continuously (C) (including two studies at rest) and 21 studies monitored T*_sk_* not continuous (N/C) (T*_sk_* at a single point).

**Overview of the T*_sk_* equipment**

- 19 studies used IRT
- Five studies used thermocouples
- Seven studies used thermistors including two studies using the i-Button^®^

**Supplementary Table S1.** **Overview of selected equine studies measuring T*_sk_* (°C) at rest (R) or during exercise (E), non-continuous data points (N/C, at a single point) or monitoring continuously (C).** Only essential data are presented. T*_sk_*: skin temperature; T_CV_: central venous blood temperature; T*_re_*: rectal temperature; T*_RA_*: right atrial blood temperature; T*_PA_*: pulmonary artery blood temperature; T*_a_*: ambient temperature; max.: maximum; min: minutes; IRT: infrared thermography; ROIs: regions of interest; *italic*: data related to the skin and coat characteristics; n =: = number of horses involved in the study; field: field studies; - : data not available; BSA: body surface area; QH: Quarter Horse; TB: Thoroughbred; BM: body mass; TX: therapy; HH, HD, CD: hot-humid, hot-dry, cool-dry T*_a_*.

|  | **Publication** | **Thermo-sensor type** | **C**  **N/C** | **T_c_ Y/N;** | **Number of locations- ROIs** | **Delta T*_sk_* exercise** | **Hottest location & conclusion** | **Exercise / Rest**  **Aim of the study**  **Number of horses (n=)** |
| --- | --- | --- | --- | --- | --- | --- | --- | --- |
| 1 | (Domino et al., 2022) | IRT camera | N/C | N | 15 | NA | horse T*_sk_* > then donkey due to difference *skin thickness* | R - compare donkey & horse *skin*; n=18 & n=16 |
| 2 | (Maśko et al., 2021) | IRT camera | N/C | N | 10 ROIs: e.g., chest, hoofs | - | No T*_sk_* difference in mares nor *coat length* | R - compare pregnant and non-pregnant mares & effect of *coat length*; n=40 mares |
| 3 | (Zielińska et al., 2021) | IRT camera | N/C | N | 1: Legs | NA | *Non-pigmented* > T*_sk_* | R - compare effect of laser TX on T*_sk_* *pigmented vs non-pigmented* |
| 4 | (Brownlow and Smith, 2021) | IRT camera | N/C | N | 3: neck, shoulder, thorax | - | > 39°C = higher risk EHI | E - **field,** post exercise T*_sk_*; n=260 |
| 5 | (Giannetto et al., 2020) | IRT camera | N/C | Y, single point T_re_ | 5 ROIs | ~5°C | T*_sk_* not aligned with T_re_ | R - assess daily rhythm; n=5 |
| 6 | (Klous et al., 2020) | i-Button^®^ & glue | C | Y: C T*_re_*  N | 2: Shoulder, rump | -3°C | Pre-cooling -> T*_re_* median 0.3°C difference & T*_sk_* mean -3°C difference | E - **field,** evaluate 8 min pre-cooling to 8.5 min eventing; n=10 eventers |
| 7 | (Meisfjord Jorgensen et al., 2020) | IRT camera | N/C | N | 10 ROIs: incl chest, hoofs | - | Abdomen and flank; individual difference | R- compare 2 seasons and *coat length*; n=21 |
| 8 | (Takahashi et al., 2020) | IRT camera | N/C:  1x prior cooling | Y: T_PA_  N – only single point | 1: left thorax | -  At T*_PA_* 42°C, mean T*_sk_* > 40-41°C | When T*_PA_* 42°C, best T*_sk_* at 17^e^ ICS > 40°C  Aim until T*_PA_* < 39°C: Shower is best | E - treadmill – run to 42°C T*_PA_* – compare difference post-cooling methods; n=5 TB |
| 9 | (Wilk et al., 2020) | IRT camera | N/C | Y, single point T_re_  N | 7 ROIs | ~ 6°C | > 20% of BM -> higher T*_sk_* | E - ridden – compare BM riders; n=12 |
| 10 | (Redaelli et al., 2019) | IRT camera | N/C | N | 7 |  | Crown T*_sk_* correlation intensity | E - correlate endurance intensity with _T_*_sk_* & stress markers; n=8 |
| 11 | (Soroko et al., 2019a) | IRT camera | N/C | N | 7 muscle regions | ~1-2°C | Higher T*_sk_* in ridden horses | E - treadmill pre & post T*_sk_* compare ridden/ non ridden & correlation blood parameters; n=9 ponies |
| 12 | (Soroko et al., 2019b) | Dynamic use IRT camera q15s | N/C | N | Saddle area: 6 ROIs |  | Saddle ‘pressure’ | E – field, saddle thermal pattern; n=18 racehorses |
| 13 | (Soroko, 2018) | Dynamic use IRT camara q15s | C | N | 4: SH, neck, croup, chest |  | Neck | E – 25 min treadmill, 15 min trot (9.1 km/h); n=5 ponies |
| 14 | (Soroko et al., 2017) | IRT camera | N/C | N | 2: joints | ~25°C and T_a_ delta ~20°C | - | R - influence of T*_a_* on T*_sk_* difference between joints; n=64 |
| 15 | (Edner et al., 2015) | Thermistors and IRT camera | N/C | N | Local T*_sk_* under blanket – IRT overall thermography | - | No T*_sk_* difference | R - magnetic blanket effect; n=10 |
| 16 | (Yarnell et al., 2014) | IRT camera | N/C | N | Semitendinosus muscle | ~20°C | T*_sk_* during Dry treadmill ~ muscle activity | E - compare water treadmill; n=8 |
| 17 | (Holcomb et al., 2013) | Thermocouple - held | N/C | Y, single point T*_re_* | 2-L+R triceps | ~1°C in sun | Sun: T*_re_* & cortisol WNL (sun 37.8°C vs 37.5°C), more sweating | R - compare T*_sk_*, sweating in shade vs non-shade; n=12 |
| 18 | (Wallsten et al., 2012) | Thermistor probes  with sensors on skin | N/C | N | 2: neck, biceps, tail | ~10-20°C | *unclipped* & blanket higher thermoregulation based on RR & T*_re_* (38.2°C) | E - ridden outside several trot/canter periods over 1000m, effect of *clipping &* blanket in cold T*_a_*; n=3 |
| 19 | (Ramey et al., 2011) | IRT camera | N/C | Y, single point T_re_  N | 2: mucous membranes, trunk | - | Significant variation in T*_sk_* readings | R - IRT vs T*_re_*; n=40 |
| 20 | (Jodkowska et al., 2011) | IRT camera | N/C | Y, single point T*_re_*  N | 36 ROIs to cover whole BSA: and 25 ROIs post- exercise | ~5°C (post exercise: 25-35°C) | Head, neck, trunk.  T*_re_* WNL | E - T_max_ on BSA compare before and after jumping; n=35 |
| 21 | (Robinson et al., 2008) | implantable  microchip |  | C at rest | 1: nuchal ligament | - | Correlation influenced by T*_a_*; only 55% sensitivity at lower T*_a_* | R - compare implant T*_sk_* to T*_re_* to detect fever;  N=52 foals, n=30 QH |
| 22 | (Simon et al., 2006) | IRT camera | N/C | N | 2: FL & HL | ~5-6°C | None – all return to base T*_sk_* in 45min | E - treadmill, determine time to return to base T*_sk_*; n=6 |
| 23 | (Morgan et al., 2002) | IRT thermometer | N/C | N | 1 | - | *Clipping* result in better heat loss | E - treadmill, effect of *coat clipping* on T*_CV_*, T*_sk_*, T*_sk_* used to calculate heat loss;  n=6 SB |
| 24 | (Geor et al., 2000) | Thermocouples with sticky tape | C | Y: T_PA_  N | 1- Shaved at thorax | ~2.5°C | No difference in delta T*_sk_* in HH, HD, CD (no T_PA_ correlation & always ~3°C difference) | E - submax. & heat storage post acclimation under HH comp to CD, HD; n=6 |
| 25 | (Marlin et al., 1999a) | Thermocouple with glue | C- not exercise! | N | 2: neck & gluteal | ~ 2-6°C | T*_sk_* significant correlation sweating onset (35°C) and rate at neck | R - adrenaline anhidrosis; n=10 compared to control |
| 26 | (Marlin et al., 1999b) | Thermistor probe | C | Y: T*_RA_*  N | 1: tail skin (T*_RA_*, T*_re_*) | ~7°C | T*_sk_* indicates sweating rate | E - treadmill training acclimation & sweating rate at T*_sk_*/T*_RA_*; n=5 |
| 27 | (Marlin et al., 1998) | Thermistor probe | C | Y: T*_RA_*  N | 2: tail skin, coat | ~5°C | Response of T*_sk_* and T*_RA_* to cooling | E - treadmill  cooling study; n=5 |
| 28 | (Marlin et al., 1996) | Thermistor probe | C | Y: T*_RA_*  Y | 1: tail skin (T*_RA_*, T_re_) | ~6°C | T*_sk_* follows T*_RA_* pattern | E - treadmill  Cool compared to hot T*_a_*; n=4 |
| 29 | (Geor et al., 1995) | Thermocouples sticky tape | C | Y: T*_RA_*  N | 1- Shaved lat thorax |  | T*_sk_* diff from T_c_ | E - response to submax. (50% Vmax) under HH comp to CD, HD; n=5 |
| 30 | (Morgan, 1995) | i-Button^®^ taped | C - at rest | N | 5 ROIs: spread over body | NA | Extra energy at lower T*_a_* | R - lab study climate demand at different T*_a_* |

**References**

Brownlow, M., and Smith, T. (2021). The use of the hand‐held infrared thermometer as an early detection tool for Exertional Heat Illness in Thoroughbred racehorses: A study at racetracks in eastern Australia. *Equine Vet. Ed.* 33, 296-305. doi: 10.1111/eve.13299

Domino, M., Borowska, M., Trojakowska, A., Kozłowska, N., Zdrojkowski, Ł., Jasiński, T., Smyth, G., and Maśko, M. (2022). The effect of rider:horse bodyweight ratio on the superficial body temperature of horse's thoracolumbar region evaluated by advanced thermal image processing. *Animals* 12**,** 195. doi: 10.3390/ani12020195

Edner, A., Lindberg, L.G., Broström, H., and Bergh, A. (2015). Does a magnetic blanket induce changes in muscular blood flow, skin temperature and muscular tension in horses? *Equine Vet. J.* 47. doi: 302-307.10.1111/evj.12291

Geor, R.J., McCutcheon, L.J., Ecker, G.L., and Lindinger, M.I. (1995). Thermal and cardiorespiratory responses of horses to submaximal exercise under hot and humid conditions. *Equine Vet. J. Suppl.* 20, 125-132.

Geor, R.J., McCutcheon, L.J., Ecker, G.L., and Lindinger, M.I. (2000). Heat storage in horses during submaximal exercise before and after humid heat acclimation. *J. Appl. Physiol.* 89**,** 2283-2293.

Giannetto, C., Arfuso, F., Giudice, E., Gianesella, M., Fazio, F., Panzera, M., and Piccione, G. (2020). Infrared methodologies for the assessment of skin temperature daily rhythm in two domestic mammalian species. *J. Therm. Biol.* 92. doi: 102677.10.1016/j.jtherbio.2020.102677

Holcomb, K.E., Tucker, C.B., and Stull, C.L. (2013). Physiological, behavioral, and serological responses of horses to shaded or unshaded pens in a hot, sunny environment1. *J. An. Sci.* 91**,** 5926-5936. doi: 10.2527/jas.2013-6497

Jodkowska, M., Oblacinska, A., Tabak, I., and Radiukiewicz, K. (2011). Differences in dietary patterns between overweight and normal-weight adolescents. *Med. Wieku Rozwoj* 15**,** 266-273.

Klous, L., Siegers, E., Van Den Broek, J., Folkerts, M., Gerrett, N., Van Oldruitenborgh-Oosterbaan, M.S., and Munsters, C. (2020). Effects of pre-cooling on thermophysiological responses in elite eventing horses. *Animals* 10. doi: 10.3390/ani10091664

Marlin, D.J., Schroter, R.C., Scott, C.M., White, S., Nyrop, K.A., Maykuth, P.L., and Harris, P.A. (1999a). Sweating and skin temperature responses of normal and anhidrotic horses to intravenous adrenaline. *Equine Vet. J. Suppl.* 30, 362-369.

Marlin, D.J., Scott, C.M., Roberts, C.A., Casas, I., Holah, G., and Schroter, R.C. (1998). Post exercise changes in compartmental body temperature accompanying intermittent cold water cooling in the hyperthermic horse. *Equine Vet. J.* 30**,** 28-34. doi: 10.1111/j.2042-3306.1998.tb04085

Marlin, D.J., Scott, C.M., Schroter, R.C., Harris, R.C., Harris, P.A., Roberts, C.A., and Mills, P.C. (1999b). Physiological responses of horses to a treadmill simulated speed and endurance test in high heat and humidity before and after humid heat acclimation. *Equine Vet. J.* 31**,** 31-42.

Marlin, D.J., Scott, C.M., Schroter, R.C., Mills, P.C., Harris, R.C., Harris, P.A., Orme, C.E., Roberts, C.A., Marr, C.M., Dyson, S.J., and Barrelet, F. (1996). Physiological responses in nonheat acclimated horses performing treadmill exercise in cool (20°C/40%RH), hot dry (30°C/40%RH) and hot humid (30°C/80%RH) conditions. *Equine Vet. J. Suppl.* 22, 70-84.

Maśko, M., Witkowska‐Piłaszewicz, O., Jasiński, T., and Domino, M. (2021). Thermal features, ambient temperature and hair coat lengths: Limitations of infrared imaging in pregnant primitive breed mares within a year. *Repro. Dom. Animals* 56**,** 1315-1328. doi: 10.1111/rda.13994

Meisfjord Jorgensen, G.H., Mejdell, C.M., and Boe, K.E. (2020). Effects of hair coat characteristics on radiant surface temperature in horses. *J. Therm. Biol.* 87**,** 102474. doi: 10.1016/j.jtherbio.2019.102474

Morgan, E.K.M. (1995). Climatic energy demand of horses. *Equine Vet. J.* 27**,** 396-399. doi: 10.1111/j.2042-3306.1995.tb04960.x

Morgan, K., Funkquist, P., and Nyman, G. (2002). The effect of coat clipping on thermoregulation during intense exercise in trotters. *Equine Vet. J. Suppl.* 34, 564-567. doi: 10.1111/j.2042-3306.2002.tb05484.x

Ramey, D., Bachmann, K., and Lee, M.L. (2011). A comparative study of non-contact infrared and digital rectal thermometer measurements of body temperature in the horse. *J. Equine Vet. Sci.* 31**,** 191-193. doi: 10.1016/j.jevs.2011.02.009

Redaelli, V., Luzi, F., Mazzola, S., Bariffi, G.D., Zappaterra, M., Nanni Costa, L., and Padalino, B. (2019). The use of infrared thermography (IRT) as stress indicator in horses trained for endurance: A pilot study. *Animals* 9**,** 84. doi: 10.3390/ani9030084

Robinson, T.R., Hussey, S.B., Hill, A.E., Heckendorf, C.C., Stricklin, J.B., and Traub-Dargatz, J.L. (2008). Comparison of temperature readings from a percutaneous thermal sensing microchip with temperature readings from a digital rectal thermometer in equids. *J. Am. Vet. Med. Ass.* 233, 613-617. doi: 10.2460/javma.233.4.613

Simon, E.L., Gaughan, E.M., Epp, T., and Spire, M. (2006). Influence of exercise on thermographically determined surface temperatures of thoracic and pelvic limbs in horses. *J. Am. Vet. Med. Assoc.* 229**,** 1940-1944. doi: 10.2460/javma.229.12.1940

Soroko, M., Howell, K., Dudek, K., Wilk, I., Zastrzeżyńska, M., and Janczarek, I (2018a). A pilot study into the utility of dynamic infrared thermography for measuring body surface temperature changes during treadmill exercise in horses. *J. Equine Vet. Sci.* 62**,** 44-46. doi: 10.1016/j.jevs.2017.12.010

Soroko, M., Spitalniak-Bajerska, K., Zaborski, D., Pozniak, B., Dudek, K., and Janczarek, I. (2019a). Exercise-induced changes in skin temperature and blood parameters in horses. *Arch. Anim. Breed* 62, 205-213. doi: 10.5194/aab-62-205-2019

Soroko, M., Zaborski, D., Dudek, K., Yarnell, K., Gorniak, W., and Vardasca, R. (2019b). Evaluation of thermal pattern distributions in racehorse saddles using infrared thermography. *PLoS One* 14**,** e0221622. doi: 10.1371/journal.pone.0221622

Soroko, M.H., K (2018b). Infrared thermography: Current applications in equine medicine. *J Equine Vet. Sci.* 60, 90-96. doi: 10.1016/j.jevs.2016.11.002

Takahashi, Y., Ohmura, H., Mukai, K., Shiose, T., and Takahashi, T. (2020). A comparison of five cooling methods in hot and humid environments in Thoroughbred horses. *J. Equine Vet. Sc.* 91. doi: 103130.10.1016/j.jevs.2020.103130

Wallsten, H., Olsson, K., and Dahlborn, K. (2012). Temperature regulation in horses during exercise and recovery in a cool environment. *Acta Vet. Scand.* 54. doi: 42.10.1186/1751-0147-54-42

Wilk, I., Wnuk-Pawlak, E., Janczarek, I., Kaczmarek, B., Dybczynska, M., and Przetacznik, M. (2020). Distribution of superficial body temperature in horses ridden by two riders with varied body weights. *Animals* 10. doi: 10.3390/ani10020340

Yarnell, K., Fleming, J., Stratton, T.D., and Brassington, R. (2014). Monitoring changes in skin temperature associated with exercise in horses on a water treadmill by use of infrared thermography. *J. Therm. Biol.* 45**,** 110-116. doi: 10.1016/j.jtherbio.2014.08.003

Zielińska, P., Soroko, M., Howell, K., Godlewska, M., Hildebrand, W., and Dudek, K. (2021). Comparison of the effect of high-intensity laser therapy (hilt) on skin surface temperature and vein diameter in pigmented and non-pigmented skin in healthy racehorses. *Animals* 11. doi: 1965.10.3390/ani11071965
